# Small wind turbines and their potential for internet of things applications

**DOI:** 10.1016/j.isci.2023.107674

**Published:** 2023-08-18

**Authors:** Hao Wang, Bendong Xiong, Zutao Zhang, Hexiang Zhang, Ali Azam

**Affiliations:** 1 School of Mechanical Engineering, Southwest Jiaotong University, Chengdu 610031, P.R. China; 2 Yibin Research Institute, Southwest Jiaotong University, Yibin 644000, P.R. China

**Keywords:** Applied sciences, Internet of Things, Energy sustainability

## Abstract

Wind energy is crucial for meeting climate and energy sustainability targets. Small wind turbines (SWTs) have gained significant attention due to their size and adaptability. These turbines have potential for Internet of Things (IoT) applications, particularly in powering large areas and low-power devices. This review examines SWTs for IoT applications, providing an extensive overview of their development, including wind energy rectifiers, power generation mechanisms, and IoT applications. The paper summarizes and compares different types of wind energy rectifiers, explores recent advancements and representative work, and discusses applicable generator systems such as electromagnetic, piezoelectric, and triboelectric nanogenerators. In addition, it thoroughly reviews the latest research on IoT application scenarios, including transportation, urban environments, intelligent agriculture, and self-powered wind sensing. Lastly, the paper identifies future research directions and emphasizes the potential of interdisciplinary technologies in driving SWT development.

## Graphical abstract

**Figure:**
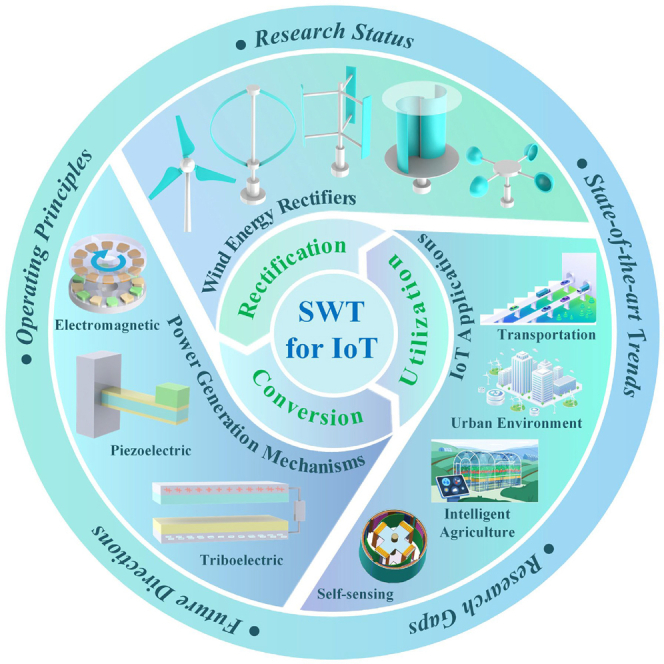

## Introduction

Energy is a ubiquitous aspect of our lives and has been the driving force behind the development of human civilization. Although exploiting fossil fuels has led to remarkable human achievements, it has also generated numerous environmental problems, notably climate change.^1^ To address this issue, the Paris Agreement states that concerted efforts are required to limit climate change this century to 1.5°C by controlling carbon emissions.^2^ One critical pathway to achieve climate goals is using clean and renewable energy sources,^3^^,^^4^^,^^5^^,^^6^^,^^7^ such as wind,^8^^,^^9^ solar,^10^^,^^11^ hydro,^12^ biomass,^13^ and geothermal energy.^14^ In recent years, wind energy^15^ has received substantial attention as it possesses several advantages, including a wide distribution, abundant capacity, and environmental friendliness.^16^

**Figure 1:**
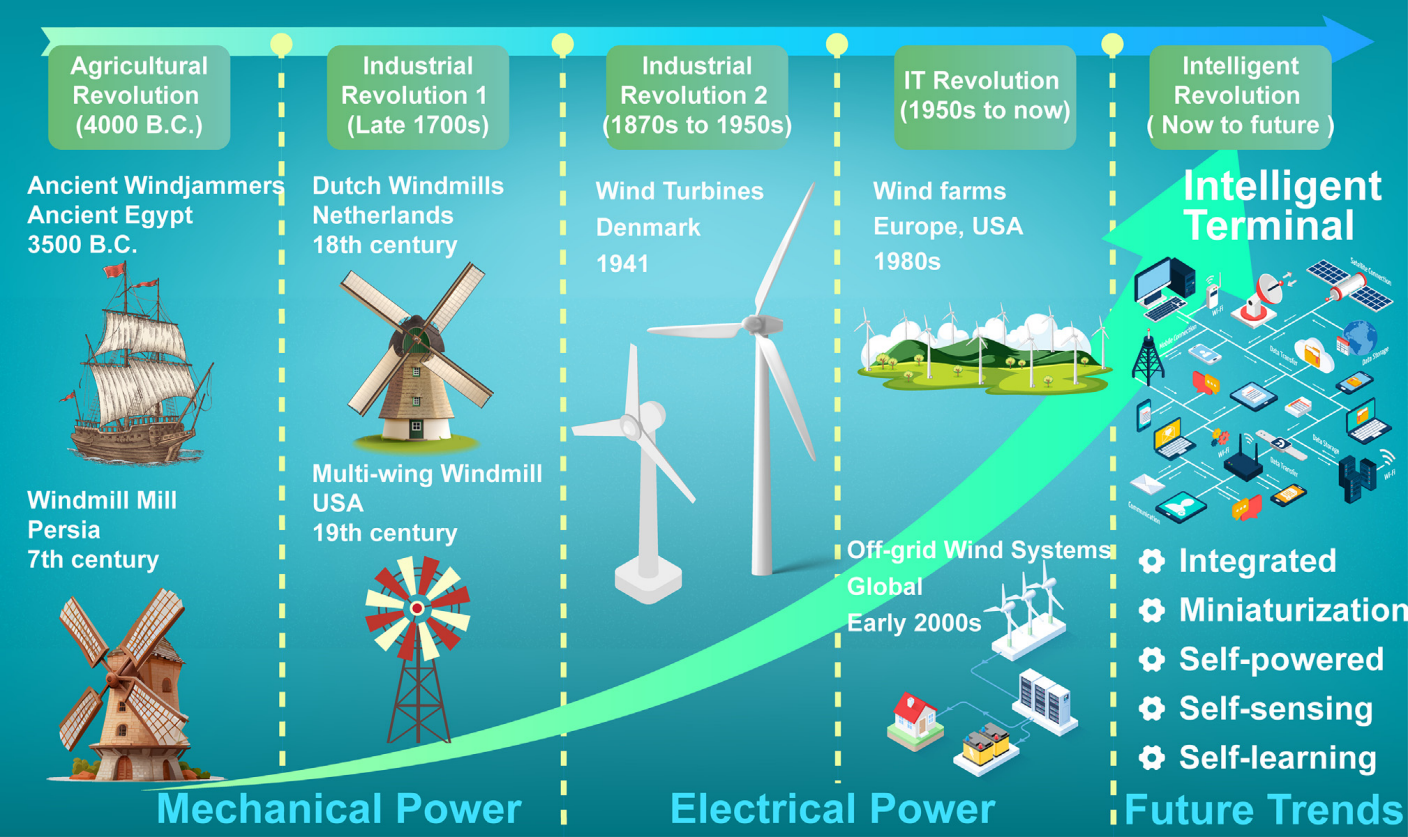
Milestones in the development of wind energy utilization technology with the productivity revolution

Wind energy, which originates from the disparity in air pressure resulting from the uneven heating of the Earth’s surface and the Coriolis effect of the Earth’s rotation, has played a crucial role in advancing human civilization,^17^ as illustrated in Figure 1. During the agricultural revolution, wind power was harnessed for sailing ships and agricultural tasks such as irrigation and milling. The first industrial revolution saw windmills being used to power factory machines like those found in textile production. With the advent of the second industrial revolution and advances in aerodynamics, wind turbines were developed, thus increasing the efficiency of wind energy and making it a viable technology.^18^ Following the information technology revolution and the oil crisis, wind energy became a significant focus of renewable energy research, leading to the creation of wind farms and commercialization. The worldwide installed wind power capacity increased from 238 GW in 2011 to 837 GW in 2021, with an average annual growth rate of 13.4%.^19^ Nevertheless, more significant efforts are necessary to accomplish the climate objectives outlined in the Paris Agreement. As the intelligent revolution progresses, wind energy harvesting technology will increasingly incorporate the Internet of Things (IoT), artificial intelligence (AI), and big data. This incorporation will result in features such as integration, miniaturization, self-powering, self-sensing, and self-learning, allowing for even more rapid development.

Historically, wind farms comprising sizable horizontal axis wind turbines have dominated the installed capacity, but their ongoing technological innovation has become further constrained. The energy harvested from wind is directly proportional to the windward area of the turbine; however, the cost of turbines escalates with their size, and increasing the power ratings simply by raising the turbine’s rotor diameter is becoming increasingly arduous.^17^ Wind farms are required to be centrally situated in geographic locations featuring less populated, more open terrain with high wind energy quality. Despite the widespread availability of wind energy, wind farms' geographic attributes dictate higher power transmission costs. Extensive technological advancements in wind energy are necessary to bridge the gap between installed wind power capacity and the vast amount of untapped global wind resources.

Energy demand is the fundamental driver of technological innovation. With the rise of information technology and IoT,^20^ small wind turbines (SWTs) have emerged as one of the most promising growth areas and offer a complementary alternative to traditional wind farms.^21^ Unlike centralized wind farms, SWT can adapt to widely distributed and ubiquitous wind resources, thanks to their flexible sizes and locations. This makes them an excellent fit for the energy needs of IoT nodes characterized by wide areas, low power consumption, and scalability.^22^^,^^23^ IoT sensors and embedded devices predominantly rely on batteries with a limited lifespan, necessitating frequent maintenance and replacement.^24^ Such processes bear significant costs while also posing environmental hazards.

**Figure 2:**
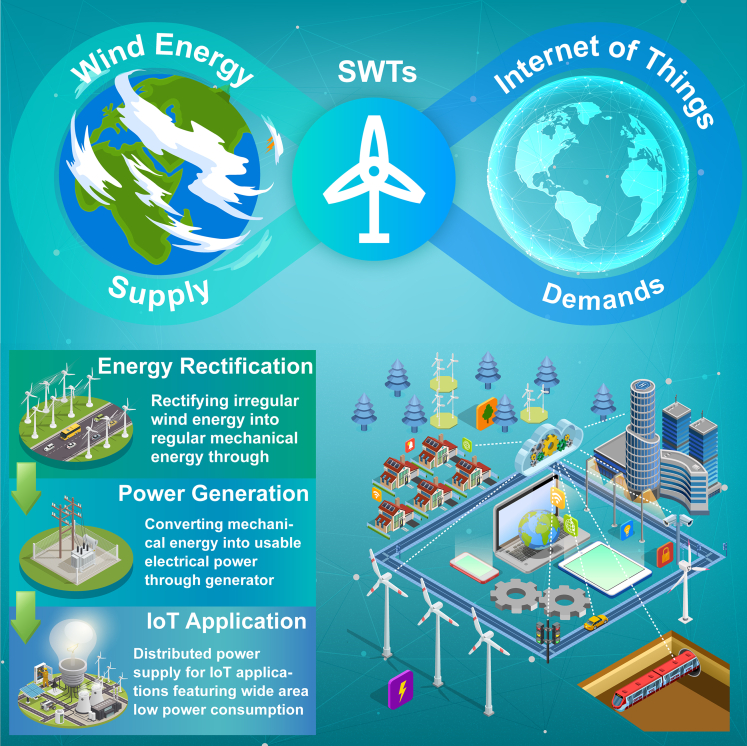
Deploying small wind turbines as a crucial and feasible measure to address the energy requirements of low-power IoT devices

Integrating SWTs into IoT holds great promise for sustainable IoT applications. Traditionally, IoT consists of a wide-area network comprising numerous low-power devices such as sensors, controllers, communicators, and memories. Consequently, a stable, reliable, and long-term energy supply solution is crucial for the future development of IoT. By addressing the energy demands associated with wide-area low-power consumption, IoT technology effectively fosters the development and commercialization of SWTs. Sustainable IoT applications offer an ideal platform for SWTs, given their immense growth potential, which, in turn, suggests significant market opportunities for SWTs. Once SWTs become the primary energy source for IoT, the resulting sustainable IoT infrastructure will no longer be constrained by energy supply limitations. As a result, the applicability and reach of IoT technology will be further enhanced, thereby maximizing its potential. Ultimately, the integration of SWTs and IoT technologies complements each other, providing an effective solution for building sustainable IoT. As highlighted in Figure 2, SWTs installed near IoT nodes capture ambient wind energy, rectify it, and generate electricity to provide a reliable energy source for IoT systems. Employing wind energy to power low-power devices significantly improves energy efficiency and reduces transmission facility costs, particularly in remote areas that lack centralized grids.

**Figure 3:**
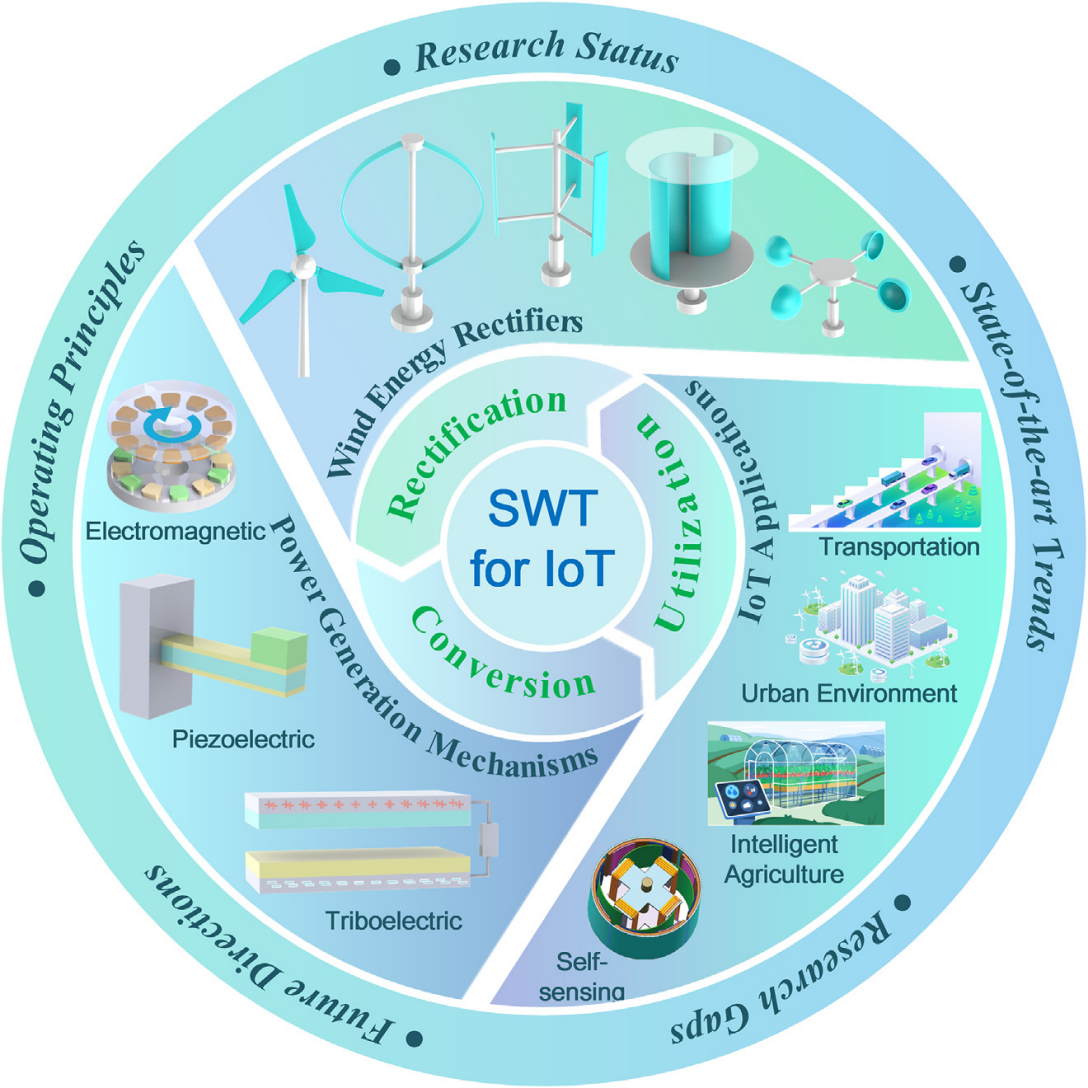
Architectural framework for the review of SWT for IoT applications

Recent years have seen important developments in the miniaturization of wind turbines and distributed wind energy harvesting.^25^^,^^26^^,^^27^ These advancements have helped to make significant strides toward achieving wind power goals, even though the basic concept of wind turbines has remained unchanged. As the field progresses rapidly, a comprehensive review of SWT for IoT applications is essential to guide future commercialization and exploration, as depicted in Figure 3. Although various technical solutions exist in terms of harvesting scope, conversion, and application targets, the wind harvesting process typically involves three stages^28^: (1) rectification, (2) conversion, and (3) utilization. When wind hits the wind rectifier, some of its kinetic energy is converted into mechanical energy in the form of rotation.^29^ Different energy conversions mechanisms, such as electromagnetic,^30^^,^^31^ piezoelectric,^32^ and triboelectric^33^^,^^34^ methods, then transform the mechanical energy into electrical energy. Finally, the generated energy is either stored or utilized directly.

This review’s innovative contribution and driving force is a comprehensive overview and critical discussion of the recent advances, research gaps, and application fields of SWT for IoT applications. The review is organized as follows: “wind energy rectifiers” covers the wind energy rectifiers (WERs) of SWTs and compares their basic principles, operating characteristics, and state-of-the-art advances. “Power generation mechanisms” presents the operating principles and characteristics of the generator system of SWTs and outlines state-of-the-art research trends. “Sustainable IoT based on SWTs” summarizes the distributed application scenarios and research progress of SWTs. In “research trends, gaps, and future directions,” research gaps and future directions are identified. The review concludes with a summary of the findings.

## Wind energy rectifiers

The essence of wind energy lies in the kinetic energy produced by large-scale airflow, which is directly proportional to the speed of the air flow.^35^ The WER plays a crucial role in the initial stage of wind energy harvesting as it extracts the kinetic energy of airflow and converts it into usable mechanical energy. The rotor,^36^ consisting of the blades, rotor shaft, and hub, serves as the WER in wind turbines in a narrower sense, determining the efficiency of wind energy harvesting and the operational safety of the turbine. This section provides an overview of the wind energy rectification process and the most recent research trends.

### The basis of wind energy rectifier

The basis of the WER lies in the rotor’s ability to capture wind energy and convert it into usable mechanical energy. Nonetheless, the way in which this conversion is achieved varies across rotors with different configurations. This section provides a comprehensive classification, operational principles, and characteristics of rotors that are essential in IoT applications.

### Classification of rotors

**Figure 4:**
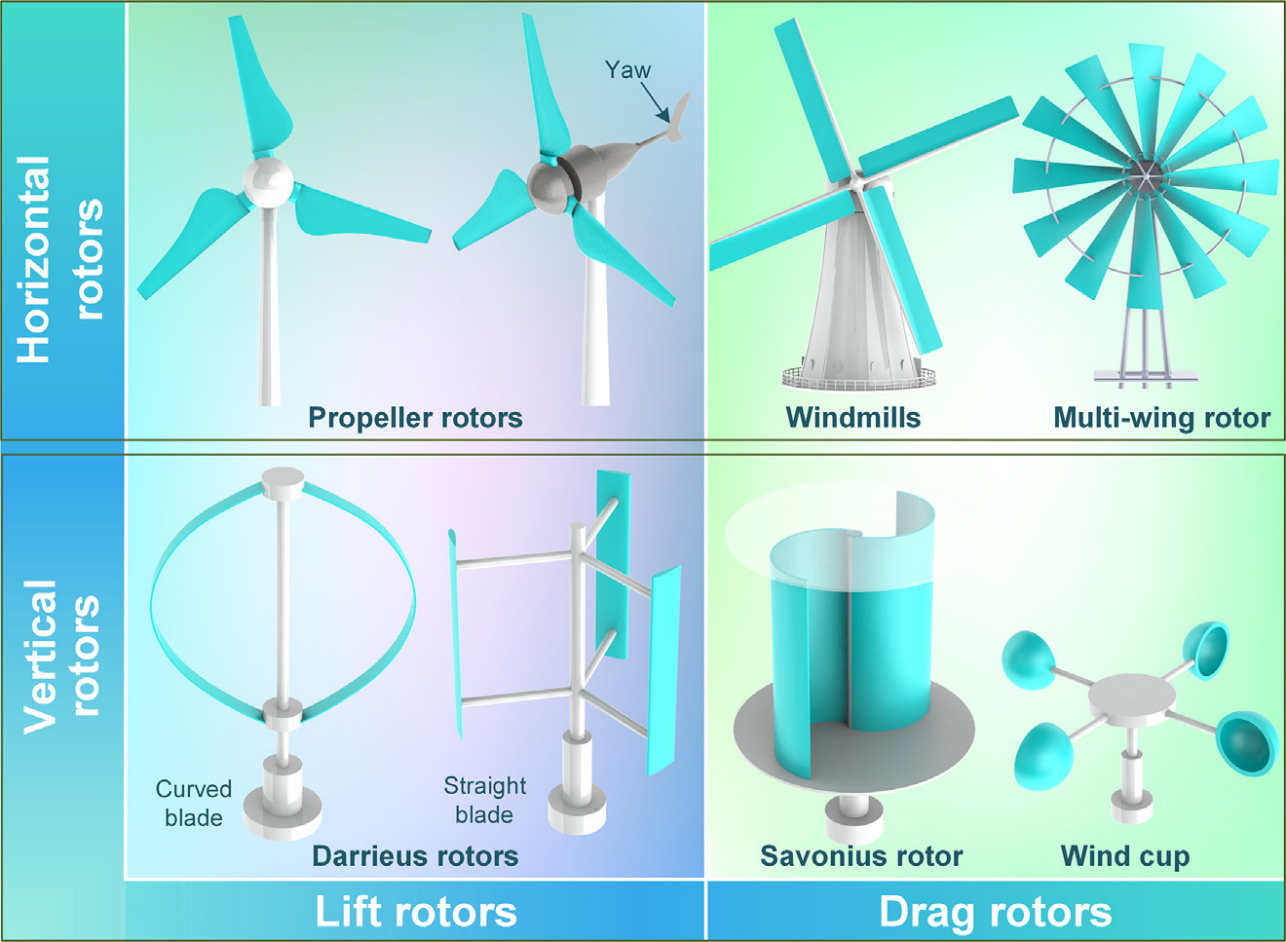
Structure and classification of rotors

As depicted in Figure 4, rotors can be classified into two mainstream types. Structurally, rotors can be categorized as horizontal or vertical based on the rotational direction of the rotor shaft. Meanwhile, regarding operational principles, rotors can be divided into lift and drag rotors, depending on the force exerted on the blades.

The horizontal rotor,^37^ characterized by a rotational axis parallel to the airflow plane, has a simple structure and impressive self-starting capability. However, to align with the wind direction, an additional yaw mechanism is needed. The horizontal axis wind turbine (HAWT) is a turbine with a horizontal axis rotor, with variants such as windmills, American multi-wing rotors, and propeller rotors.^38^^,^^39^ Propeller rotors, mainly utilized in commercial wind turbines using the latest aerospace technology, have high rotation speeds and excellent performance, making them the primary form of small HAWTs in recent years.^40^ Similarly, vertical rotors, characterized by a rotation axis perpendicular to the airflow plane, have a compact omnidirectional structure and consist mainly of Darrieus, Savonius, and wind cup turbines.^41^ The Darrieus rotor, invented by French engineer G. J. M. Darrieus in 1931, has been optimized for practical applications.^42^ This rotor can be further categorized into curved and straight blades, based on the blade structure. The curved blade is designed for pure tension, without any centrifugal load on the blade. The straight blades are relatively cheaper to manufacture, but a robust support structure is necessary to overcome the bending stresses caused by centrifugal forces.^43^ Moreover, the Savonius rotor, invented by Finnish engineer Sigurd Johannes Savonius in 1922, usually operates with two to three curved blades that resemble the letter S. This type of rotor operates via the wind resistance difference produced by the airflow through the curved blades in distinct directions and has a large starting torque, low working wind speed, and simple construction.^44^ In addition, the wind cup, a specific kind of Savonius rotor with three to four spoon-shaped blades, is typically utilized as an anemometer and recently utilized as a rotor for wind energy rectification in some micro wind harvesting devices.^45^

### Operating principle

Rotors can be classified into lift and drag types based on their operating principle.^46^ The lift type comprises the horizontal rotor and Darrieus rotor, which have blades with airfoil profiles that operate using the lift, generated by the airfoil’s aerodynamic properties. In contrast, the drag type primarily comprises the Savonius rotor, characterized by curved blades that operate using the drag difference created by the alteration in the blades' curvature.

The blade element theory^47^ is a standard method used to study the aerodynamic performance of rotors, and their basic operating principles are based on this concept. According to this theory, the blade is divided into microcells, each serving as an individual blade element. These blade elements are then integrated along the spanwise direction, and the critical aerodynamic parameters, such as the blade and rotor torque output, are calculated.

**Figure 5:**
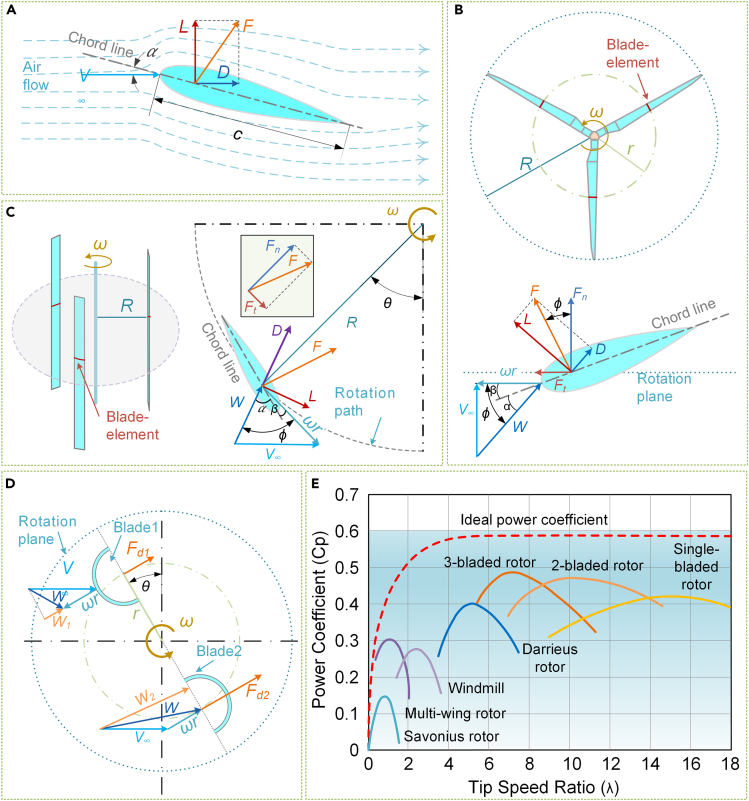
Operating principles of different rotors (A) Forces on the blade: lift and drag. (B) Horizontal rotor. (C) Darrieus rotor. (D) Savonius rotor. (E) Distribution of power coefficient for different rotors.

Bernoulli’s principle states that the difference in flow velocity on the blade surface caused by a specific airfoil profile will result in a fluid motion that generates a reaction force *F*. This reaction force can be deconstructed into two components: the lift force *L*, which acts perpendicular to the direction of the incoming velocity *V*_∞_, and the drag force *D*, which acts parallel to the direction of *V*_∞_, as revealed in Figure 5A. The Kutta-Joukowski theorem sets the relationship between the lift force *L* and drag force *D* acting on the blade as the equation shown below:

(Equation 1) $$L=\frac{1}{2}\rho c{V_{\infty }}^{2}C_{l}$$

(Equation 2) $$D=\frac{1}{2}\rho c{V_{\infty }}^{2}C_{d}$$

where, *ρ* is the air density, *c* is the blade chord length, and *C*_*l*_ and *C*_*d*_ are the lift and drag coefficients of the airfoil, respectively.

The torque that the airflow force *F* generates on the blades differs between horizontal and vertical rotors, depending on the differences in their structure and layout. In the case of the horizontal rotor, as presented in Figure 5B, the blade element’s thickness of *dh* is selected at a distance *r* from the rotating axis. The relative wind speed *W*, at this blade element, equals the sum of the incoming speed *V*_∞_ and the tangential velocity *ωr*. The following equation describes this relationship:

(Equation 3) $$W=\sqrt{{V_{\infty }}^{2}+\omega^{2}r^{2}}$$

where *ω* is the rotor angular velocity.

The angle between the relative wind speed and the chord of the blade (i.e., inflow angle) is

(Equation 4) $$\varphi =\arctan\left(\frac{V_{\infty }}{\omega r}\right)=\arctan\left(\frac{1}{\lambda }\right)$$

where, *λ* is the tip speed ratio (TSR) of the blade, which is defined as

(Equation 5) $$\lambda =\frac{\omega r}{V_{\infty }}$$

The angle of attack at the position of the leaf element is

(Equation 6) $$\alpha =\varphi -\beta =\arctan\left(\frac{V_{\infty }}{\omega r}\right)-\beta =\arctan\left(\frac{1}{\lambda }\right)-\beta$$

where *β* is the pitch angle at the blade element.

At the blade element, the lift *L* and drag *D* can be decomposed into a force *F*_*n*_ perpendicular to the plane of the rotor and a force *F*_*t*_ tangential to the plane of the rotor, expressed as follows:

(Equation 7) $$F_{n}=L\;\cos\;\varphi +D\;\sin\;\varphi$$

(Equation 8) $$F_{t}=L\;\sin\;\varphi -D\;\cos\;\varphi$$

The torque that drives the rotor operation generated by the airflow is

(Equation 9) $$dM=NF_{t}rdr$$

The blade element analysis of the vertical rotor is shown in Figure 5C. Compared with the horizontal rotor, the inflow angle of the Darrieus rotor is expressed as

(Equation 10) $$\varphi =\arctan\left(\frac{V_{\infty }\;\sin\;\theta }{\omega r-V_{\infty }\;\cos\;\theta }\right)=\arctan\left(\frac{\sin\;\theta }{\lambda -\cos\;\theta }\right)$$

The lift force *L* and drag force *D* on the blade are still decomposed into normal force *F*_*n*_ and tangential force *F*_*t*_, whose expressions are shown in Equations 7 and 8. The operating torque can be calculated by

(Equation 11) $$dM=NF_{t}Rdh$$

The Savonius rotor uses the drag generated by the airflow disparity on diverse surfaces of the curved blades, as portrayed in Figure 5D. As such, the rotation is solely due to the action of the drag. At an azimuth of *θ*, the rotor can use the tangential segments of the relative wind speed *W*, located on both its convex blade side (blade 1) and concave blade side (blade 2). The following equations represent these segments:

(Equation 12) $$W_{1}=V_{\infty }\;\cos\;\theta -\omega r$$

(Equation 13) $$W_{2}=V_{\infty }\;\cos\;\theta +\omega r$$

where *ω* is the rotor angular velocity and *r* is the radius at the center of mass of the blade.

The corresponding thrusts on the convex and concave surfaces are

(Equation 14) $$F_{d1}=\frac{1}{2}\rho c{\left(V_{\infty }\;\cos\;\theta -\omega r\right)}^{2}C_{d1}$$

(Equation 15) $$F_{d2}=\frac{1}{2}\rho c{\left(V_{\infty }\;\cos\;\theta +\omega r\right)}^{2}C_{d2}$$

where *c* is the blade chord length and *C*_*d1*_ and *C*_*d2*_ are the drag coefficients of the convex and concave surfaces, respectively.

The net thrust of the rotor is

(Equation 16) $$F_{d}=F_{d2}-F_{d1}$$

The working torque acting on the rotor shaft is

(Equation 17) $$dM=F_{d}rdh$$

### Comparison and discussion

The horizontal and Darrieus rotors employ the lift force of the airflow acting on the blades to operate the rotor. However, while the attack angle of the horizontal rotor blades stays constant at a constant incoming wind speed and rotation speed, it varies cyclically with the blade azimuth on the Darrieus rotor. This suggests that the horizontal rotor blades are subjected to moderate load fluctuations. As a result, the blades' longevity is maintained. For the horizontal rotor, the torque from airflow acting on it depends on its spreading radius. Conversely, for the vertical rotor, the torque is based on its support radius. Therefore, the Darrieus rotor can accumulate more energy by increasing the structure’s size in a limited space. In contrast, the Savonius rotor operates using the drag difference, and it features a compact structure with a large windward region. Consequently, it has an excellent self-starting capability and is highly adaptable even in lower wind speed ranges.

The power coefficient (Cp) is a significant parameter that determines the aerodynamic characteristics of a rotor. It measures the rotor’s ability to convert mechanical energy captured from the airflow to kinetic energy of the air flowing through the rotor per unit time.^48^ According to Betz, a German aerodynamicist, a rotor’s Cp has a maximum value of 0.593, which is called the Betz limit.^49^ The Cp curve^50^ as a function of blade TSR for different rotors is presented in Figure 5E. The lift rotor has a high Cp, with the three-blade horizontal rotor and Darrieus rotor having Cp values ranging from 0.33 to 0.48 and 0.28 to 0.40, respectively, indicating significant potential for power generation. However, to achieve this high performance, the rotor must operate at a higher TSR, which can pose challenges to rotor stability and wind energy quality and may underperform under low wind speed conditions. On the other hand, the Savonius rotor, with its lower power coefficient range, performs well in low wind speed environments or low power consumption devices such as anemometers. Its self-starting capability, an advantage of the drag-type rotor, and the wide range of operating wind speeds contribute to its excellent performance. Table 1 summarizes the structure, advantages, limitations, and application range for each rotor type. These factors should be considered in wind energy harvesting for IoT applications.

**Table 1:** Structure, advantages, limitations, and range of application for rotors

Category	Structure	Advantages	Limitations	Applications
Horizontal rotor	• Lifting airfoil blade • Rotation axis parallel to wind direction • With yaw mechanism	• High wind energy utilization • High starting torque, easy to start • Low torque pulsation, high reliability	• Wind direction dependency • Low space utilization • Complex blade design and manufacturing with high production costs	• Low-mid-high wind speed ambient • High power demands • High-reliability requirements
Darrieus rotor	• Lifting airfoil blade • The rotor axis perpendicular to wind direction • Yaw mechanism absence	• High wind energy utilization • Omni-directional design: no requirement to face the wind • Compact rotor structure: high space utilization	• Low starting torque and difficulty with self-start • High torque pulsation and parts fatigue • High operating speed, reliability reduced	• Mid-high wind speed ambient • Space size limitation • Mid-high power demands
Savonius rotor	• Non-lifting airfoil blade • The rotor axis perpendicular to the wind direction • Yaw mechanism absence	• High starting torque, easy to start • Omni-directional, requiring no facing into the wind • Simple structure, low production cost	• Low wind energy utilization • Slow rotor speed, low power generation potential	• Low wind speed ambient • Low noise level • High-reliability requirements

### Cutting-edge research trends

Several research endeavors have been conducted to enhance rotors starting and operating performance, such as blade design, rotor optimization, and other configurations. This section outlines the cutting-edge research trends for each rotor type separately.

#### Horizontal rotor

**Figure 6:**
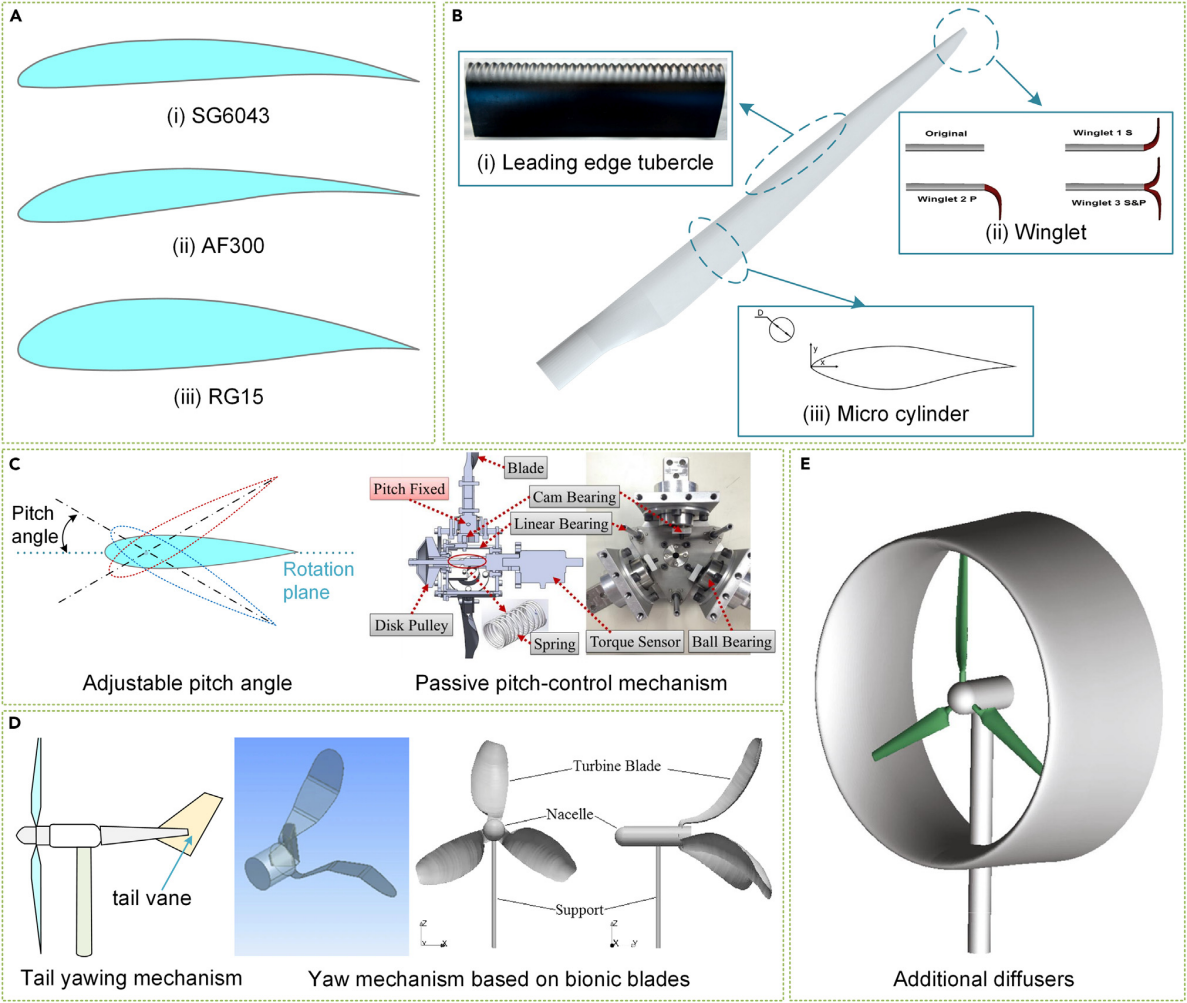
State-of-the-art research trends in horizontal rotors (A) Airfoil design for low Reynolds number. (B) Blade design technologies.^58^^,^^65^^,^^71^ (Elsevier Publishing.^58^^,^^65^^,^^71^ Reproduced with permission. All rights reserved). (C) Variable pitch^82^ (MDPI Publishing.^82^ Reproduced with permission. All rights reserved). (D) Passive yaw mechanisms.^90^^,^^91^(Elsevier Publishing.^90^ Springer Publishing.^91^ Reproduced with permission. All rights reserved). (E) Additional horizontal rotor diffusers.^94^(Elsevier Publishing.^94^ Reproduced with permission. All rights reserved).

The blade is a crucial component that interacts directly with airflow, and well-designed blades enhance the efficiency of small horizontal rotors. Standard low Reynolds number airfoils,^51^ such as the SG604X series,^52^ AF300,^53^ and RG15 series^54^ shown in Figure 6A, have been developed over the past decade. Airfoil optimization aims to enhance the lift-to-drag ratio, particularly in low Reynolds numbers and low wind environments. To further improve rotor aerodynamics, leading-edge tubercles, winglets, and miniature cylinders have been recently developed, as depicted in Figure 6B.

Bionic-based leading edge tubercles^55^ significantly enhance blade stall characteristics and are popular among researchers.^56^^,^^57^^,^^58^ Huang et al.^59^ experimented with different amplitudes and wavelengths of leading-edge tubercles on small horizontal rotor blade systems and found that these modifications effectively delayed the stall speed, improving performance at low TSR. Bai et al.^60^ conducted further experimental research and discovered that leading-edge sinusoidal tubercles significantly improved the power coefficient of rotors. Similarly, Cai et al.^61^ investigated the effects of a single tubercle on the performance of a two-dimensional airfoil and found that the modified airfoil had a unique two-step stall performance compared with the sudden stall of the standard airfoil. Recent numerical studies by Ke et al.^62^ revealed that leading edge tubercles lower the onset of blade stall, particularly at a high head angle and low TSR, resulting in enhanced aerodynamic performance.

Adding a winglet to the blade tip is an effective method of increasing the power of horizontal rotors. They suppress tip vortices, minimizing drag and improving the lift-to-drag ratio of the wing, leading to greater operational stability.^63^ Tobin et al.^64^ discovered an 8.2% improvement in power coefficient and a 15% increase in moment coefficient with winglets in their wind tunnel experiment using 0.12-m-diameter rotors. Bidirectional winglets proposed by Zhu et al.^65^ generated more power output than unilateral winglets at various blade pitch angles and TSRs, as presented in Figure 6Bii. Farhan et al.^66^ introduced rectangular winglets on S809 blades, which significantly improved rotor performance. In a recent study, Garcia-Ribeiro et al.^67^ compared 12 winglet configurations to better understand the effect of winglet parameters on rotor efficiency.

The micro-cylinder technique is a passive flow control method that improves blade aerodynamic performance without altering its structure.^68^ Huang’s team discovered a maximum increase of 27.3% in blade torque by placing micro-cylinders at the leading edge of the blade.^69^ They further researched optimal micro-cylinder placement, considering the relative inflow angle and exploring the mechanism for improving aerodynamics.^70^ Huang’s team also compared the effects of oscillating and static micro-cylinders with bare blades on an S809 airfoil using flow field analysis.^71^^,^^72^ Mostafa et al.^73^ quantitatively analyzed the impact of micro-cylinders on small horizontal rotor performance, revealing the effect of different micro-cylinder parameters on power generation. To improve blade performance in low Reynolds numbers and low wind operations, various optimization techniques aim to reduce or slow down the efficiency loss caused by blade stalls.

Rotor design greatly affects horizontal rotor performance, with various studies showing three-blade propeller-type rotors to be superior.^74^^,^^75^^,^^76^^,^^77^ As research on small horizontal rotors advanced, the significance of pitch angle was discovered.^78^^,^^79^^,^^80^ Mayer et al.^81^ analyzed how different pitch angles impacted small horizontal rotor starting performance. While an increased pitch angle enhances starting capability, it reduces potential power generation at high wind speeds. Fixed pitch angles cannot accommodate wind speed variability, causing small horizontal rotors to employ pitch mechanisms to overcome this limitation. Chen et al.^82^^,^^83^ created and tested a passive pitch control system utilizing a centrifugal drive mechanism to stabilize output power, as seen in Figure 6C. Shuwa et al.^84^ conducted wind tunnel tests to comprehensively analyze a Watt centrifugal governor-based pitch angle adjustment mechanism.

Rotational alignment is critical for horizontal rotors, yet mimicking the intricate yawing methods used by larger horizontal rotors proves challenging for smaller versions. Recently, researchers have turned to simple passive yawing mechanisms suited for small horizontal rotors.^85^^,^^86^ Narayana et al.^87^ modeled yaw motion with wind vane dynamic response and alternative yaw dynamic equations. Based on field measurements, Evans et al.^88^ devised ways to diminish yaw error by evaluating small horizontal rotor yaw dynamics. Wu et al.^89^ studied dynamic yaw’s effect on rotor wake, revealing variations in the rotor’s overall aerodynamic performance. As depicted in Figure 6D, Chu^90^^,^^91^ suggested two biologically inspired blades that permit the rotor to face the wind’s direction, unlike conventional tail vane yaws. When designing the rotor, it is essential to consider parameters like blade count, mounting angle, and yaw mechanism to maximize wind energy harvesting efficiency with a logical layout.

Utilizing additional diffusers to boost the local flow rate proves an effective means of significantly enhancing small horizontal rotor power output.^92^^,^^93^ Bontempo and Manna^94^ established, as demonstrated in Figure 6E, that a ducted rotor achieved superior flow quality and collection efficiency compared with an open one. Kosasih and Saleh Hudin’s research confirmed that diffusers remained functional in highly turbulent, high-degree-of-freedom environments.^95^ In addition, a series of studies^96^^,^^97^^,^^98^^,^^99^^,^^100^ analyzed how diverse geometric parameters impacted the power gain effect and offered practical application guidance for diffusers.

#### Darrieus rotor

**Figure 7:**
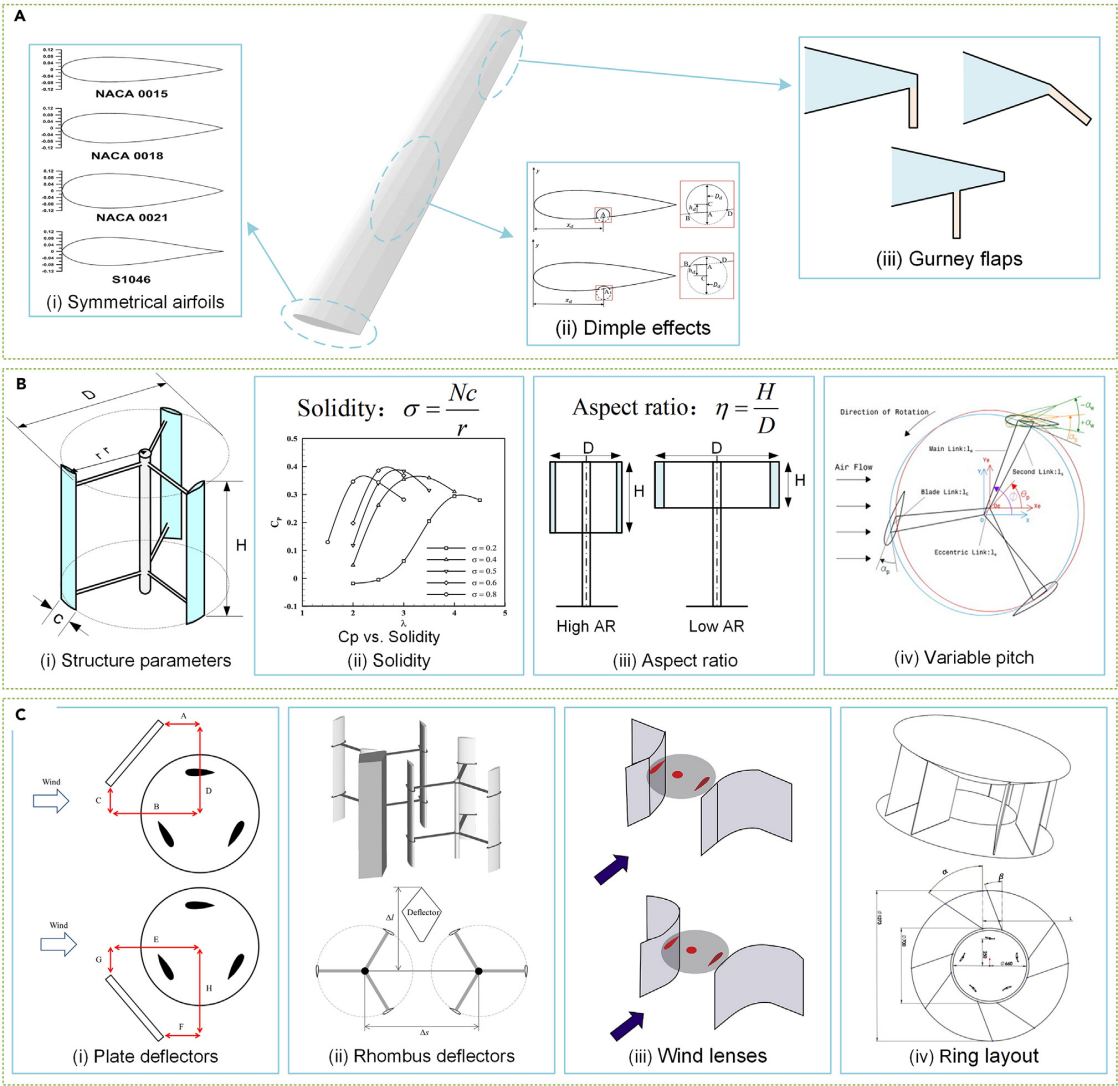
State-of-the-art research trends in Darrieus rotors (A) Blade design for vertical rotors.^102^^,^^113^^,^^118^(Elsevier Publishing.^102^^,^^118^ Taylor & Francis Publishing.^113^ Reproduced with permission. All rights reserved). (B) Rotor design and optimization technologies.^105^^,^^126^^,^^129^ (Elsevier Publishing.^105^^,^^126^ Springer Publishing.^129^ Reproduced with permission. All rights reserved). (C) Flow enhancement devices.^144^^,^^146^^,^^147^^,^^153^ (Elsevier Publishing.^144^^,^^146^^,^^147^^,^^153^ Reproduced with permission. All rights reserved).

Like horizontal rotors, Darrieus rotors use lift forces generated by the blades. Studies have identified suitable airfoil types,^101^^,^^102^^,^^103^ including symmetrical airfoil types like the NACA 00XX series, due to their favorable performance as the blade transitions between pressure and suction sides with azimuthal changes,^104^^,^^105^^,^^106^ as depicted in Figure 7Ai. Similar optimization methods used for small horizontal rotors, such as low Reynolds number airfoils,^107^ leading-edge tubercles,^108^ winglets,^109^ and micro cylinders,^110^ can also benefit Darrieus rotors. Furthermore, specific blade optimization techniques like dimples^111^ and Gurney flaps^103^ have been designed for the Darrieus rotor.

The dimple cavity design feature enhances aerodynamic performance by passively controlling flow on the blade surface, achieved by removing portions of the blade’s airfoil shape.^111^ Sobhani et al.^112^ determined that circular cavities located on the blade’s surface provide the most improved performance. Another study, conducted by Yoo and Oh^113^ on vertical axis wind turbines (VAWTs), examined how dimple location, size, and depth affected performance, using artificial neural network and genetic algorithms to optimize the dimple configuration, as illustrated in Figure 7Aii. Yousefi et al.^114^ analyzed dimple layout’s effect on rotor performance, concluding that a single dimple airfoil surpassed double dimple alternatives. Finally, Ibrahim et al.^115^ discuss active control techniques incorporating dimples and blowers, reporting significant improvements in self-start capability. Gurney flaps^116^ are small flat plates mounted vertically on the airfoil’s trailing edge, as depicted in Figure 7Aiii, to passively regulate flow control. Zhu et al.^117^ investigated the impact of Gurney flap geometric parameters on rotor performance, while Syawitri et al.^118^ studied their ideal mounting angle and height. Liu et al.^119^ utilized an active flow control method integrating dimple and Gurney flaps. Airfoil optimization and flow control are fundamental to lift blade design for small horizontal and Darrieus rotors. Researchers have recently focused on developing and optimizing flow control techniques to improve blade aerodynamic performance.

The Darrieus rotor still lacks consistency in research despite recent development.^120^ The Darrieus rotor has several key geometric features, including solidity,^121^ aspect ratio,^122^ and pitch angle,^123^ all of which are illustrated in Figure 7B. Solidity, which is defined as the ratio between the blade area and swept rotor area, has a significant impact on the rotor’s starting capability, power coefficient, and TSR range. Studies indicate that high solidity helps the rotor to self-start but peak performance is reduced due to dynamic stall caused by blade interactions.^124^^,^^125^ There is an ongoing debate about the appropriate range of solidity, with some researchers suggesting 0.4–0.6,^126^ others recommending 0.2–0.4,^127^ and others maintaining that 0.25–0.5^128^ is ideal. Additional variables, such as Reynolds number, rotor size, and wind speed range, also impact density selection, requiring further discussion.

Aspect ratio refers to the ratio of blade height to rotor diameter. An earlier study^129^ proposed that, for a given swept wind area, a lower aspect ratio should be selected to enable longer blade chord lengths, which would increase the chord Reynolds number and improve performance. However, a different study^130^ discovered that higher aspect ratios could reduce blade tip losses and improve rotor performance more effectively than the Reynolds number effect. This finding has gradually become the dominant viewpoint in the field.^125^^,^^131^^,^^132^ In addition, Peng and colleagues conducted wind tunnel tests that demonstrated that high aspect ratios significantly increased rotor power coefficient and TSR in turbulent and advective conditions.^133^ However, high aspect ratios entail greater strength requirements for the blades and hubs, necessitating further consideration of the optimal aspect ratio interval.

The pitch angle is a crucial parameter for rotor design. While researchers have emphasized its value in enhancing rotor performance, the optimal values vary significantly. For rotors featuring NACA0015 airfoils, optimal pitch angles in different studies have been reported as −6°,^134^^,^^135^ −4°,^136^ −2°,^137^ and 3°.^138^ The aerodynamic mechanism underlying the pitch angle’s potential to improve rotor performance requires further exploration to guide optimal pitch angle design.^139^ Variable pitching technology is a focus of research, with some scientists utilizing neural network-based control techniques to actively manipulate pitch angle and increase power output by 25% for each TSR.^140^ Several studies^141^^,^^142^ have exemplified the generic development process of active pitch technology. Although some studies have suggested that aspect ratio and solidity affect power performance, other research has indicated that pitch angle has the greatest impact on the outcome.^133^^,^^143^

Airflow-enhancing devices can boost the power of Darrieus rotors by improving incoming airflow quality. Several enhancements have been explored, including plate deflectors,^144^^,^^145^ rhombus deflectors,^146^ wind lenses,^147^^,^^148^ air ducts,^149^ and diffusers^150^ (Figure 7C). While horizontal rotors generally rely on a single form of ducted diffusers, enhancement devices for vertical axis turbines vary in structure. The flat deflector has received significant attention due to its simplicity, and studies^144^^,^^151^ have explored the impact of geometric parameters and position on the rotor. Since wind lenses, ducts, or diffusers require wind alignment, their gain effect on the omnidirectional Darrieus rotor is restricted.^152^ Consequently, the ring layout of deflectors is more appropriate for the omnidirectional rotor.^153^

#### Savonius rotor

**Figure 8:**
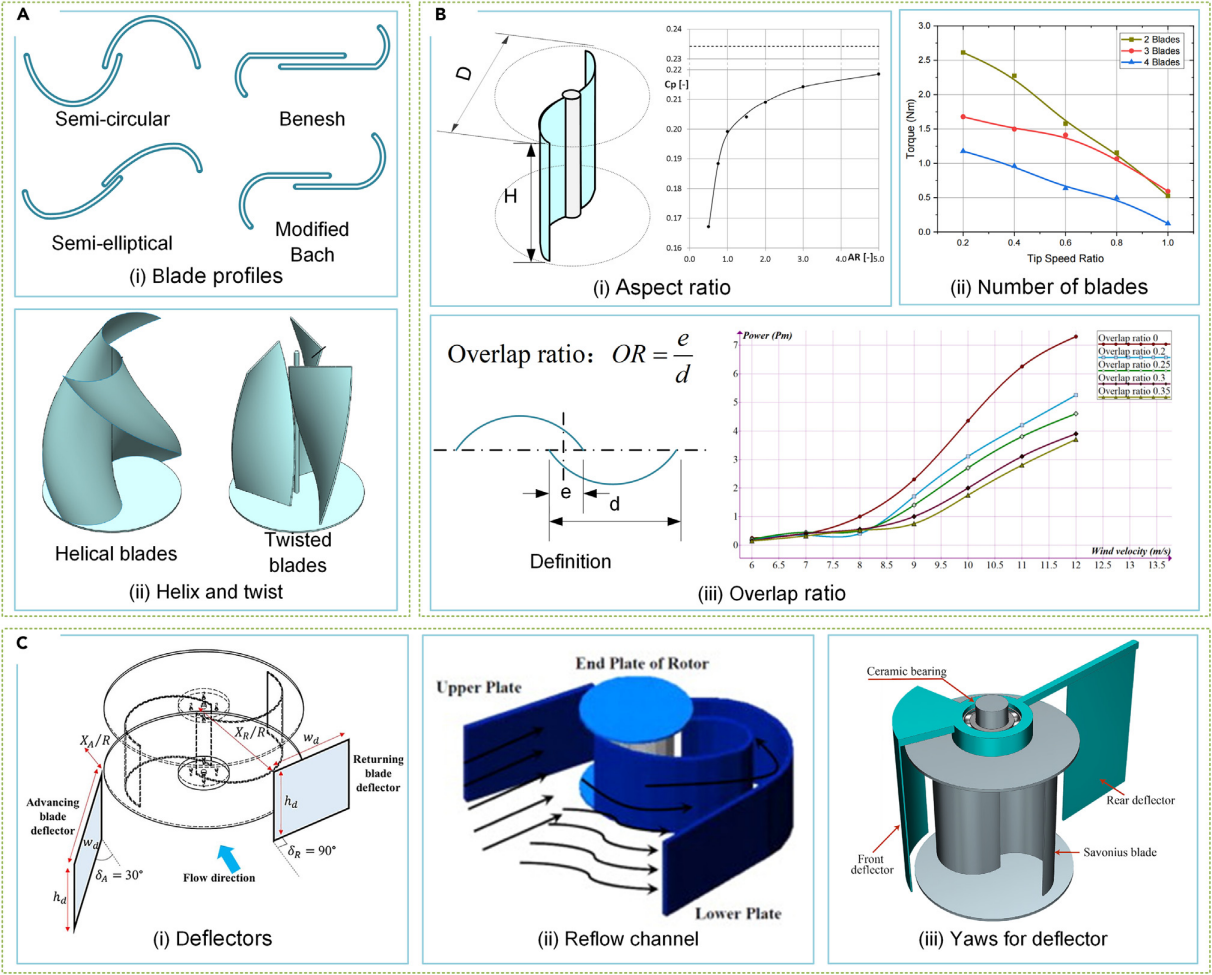
State-of-the-art research trends in Savonius rotors (A) Blade design for Savonius rotors.^158^^,^^163^ (Elsevier Publishing.^158^^,^^165^ Reproduced with permission. All rights reserved). (B) Rotor design and optimization technologies.^166^^,^^167^^,^^170^(Semarak Ilmu Publishing.^167^ MDPI Publishing.^170^ IOP Publishing.^166^ Reproduced with permission. All rights reserved). (C) Flow enhancement devices.^172^^,^^176^^,^^177^ (Elsevier Publishing.^172^^,^^176^^,^^177^ Reproduced with permission. All rights reserved).

The Savonius rotor is a reliable and cost-effective design. Current research on Savonius rotors concentrates on blade design,^154^ geometry parameters,^155^ and flow enhancement devices.^156^ Research has shown that blade design is the primary factor affecting rotor performance.^157^ Blade profiles such as semi-circular, semi-elliptical, Benesh, and modified Bach types are the traditional design focus, as shown in Figure 8Ai. Researchers have successfully created improved blade profiles, with Roy and Saha’s Benesh and Bach-based blade design performing substantially better than standard blades.^158^^,^^159^ Further studies have explored the impact of various blade profiles on rotor performance in different environments.^160^ Another research focus is on reducing torque fluctuations in the Savonius rotor using twisted or helical blades, as illustrated in Figure 8Aii.^161^^,^^162^^,^^163^ Lee et al.^164^ found that a spiral angle of 45° produces the maximum power factor, and Saad et al.^165^ created a multi-stage twisted-blade Savonius rotor to investigate the function of multiple stages in twisted blades.

An extensive study on Savonius rotors has been conducted to evaluate various geometric parameters, such as aspect ratio, number of blades, and overlap ratio, as presented in Figure 8B. Higher aspect ratio leads to better performance but requires a stronger structure, similar to Darrieus rotors.^163^^,^^166^ The excellent self-starting feature of Savonius rotors eliminates the need for additional blades to increase torque.^167^ Cost and interference can be reduced by limiting the number of blades to 2–3, while the overlap ratio, which determines the airflow in the blade gap, can generate extra thrust at the concave part of the blade.^168^ The ideal overlap ratio varies from 0.15 to 0.20 and can be affected by the environment and other structural parameters.^163^^,^^169^^,^^170^^,^^171^

Flow enhancement devices have a critical role in enhancing the Savonius rotor’s performance. These devices include deflectors,^172^ wind lenses,^173^ air ducts,^174^ and diffusers,^175^ as depicted in Figure 8C. The return blade’s negative torque poses a challenge to the rotor’s performance, which necessitates airflow guidance to the forward blade and shielding the return blade. Therefore, a deflector with curved channels has been designed by El-Askary et al.^176^ to guide airflow and shield the negative torque while increasing the positive torque, as shown in Figure 8Cii. Furthermore, Guo et al.^177^ proposed a passive alignment system that shields the return vane continuously, utilizing the deflector (Fig. 8C-iii).

## Power generation mechanisms

**Figure 9:**
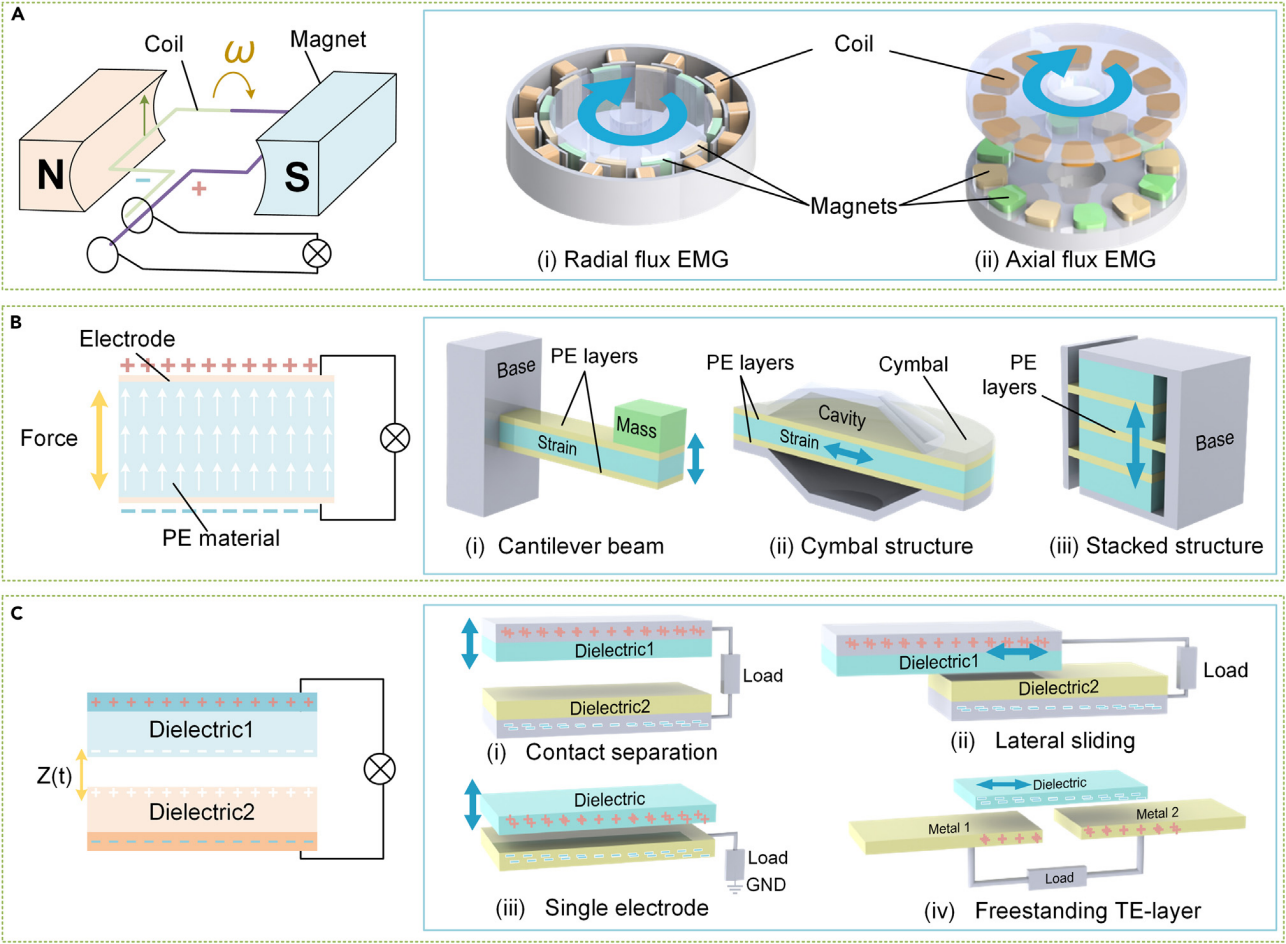
Mechanisms and structures of power generation (A) Electromagnetic effect. (B) Piezoelectric effect. (C) Triboelectric effect.

The rapid development of IoT has led to the miniaturization, intelligence, and low-power consumption of node devices.^178^^,^^179^ Power generation mechanisms (PGMs) serve as a connection between WERs and IoT devices by converting mechanical energy into electrical energy. Three main PGMs are utilized: electromagnetic (EM), piezoelectric (PE), and triboelectric (TE) methods, as illustrated in Figure 9. Researchers have realized that, while electromagnetic effects are ideal for large wind turbines due to their compatibility with high frequencies and macroscopic scales, nodes in the IoT are typically not located in high wind regions.^180^^,^^181^ Therefore, more attention has been given to the microscale PE and TE effects.^182^^,^^183^ The appropriate PGMs depend on the size, layout, and application of the SWT. This section examines the operating principles and characteristics of the three recommended PGMs, followed by an overview of research trends.

### The basis of power generation mechanisms

#### Electromagnetic effect

Michael Faraday discovered the phenomenon of EM induction and established its law, which states that a changing magnetic flux produces an electric force in a closed circuit. This force generates an induced voltage, expressed as

(Equation 18) $$\varepsilon_{V}=-N\frac{d\Phi_{B}}{dt}$$

where *N* is the turns of the coil, *Φ*_*B*_ is the magnetic flux, and *t* is the time.^184^

External mechanical excitation can generate electrical energy through the principle of electromagnetic induction. The EM generator (EMG) was designed based on this principle to convert mechanical energy into power.^185^ The rotating EMG for SWTs has two components: the permanent magnet and the coil, which create radial flux and axial flux EMG structures (Figure 9A).^186^ Both structures consist of a stator and rotor, with the magnet acting as the rotor and the coil acting as the stator for electrical energy transmission. However, EMG generation capacity is highly dependent on frequency and size and is typically recommended for macroscopic or high-frequency harvesting devices.^187^^,^^188^ Therefore, the undersized EMGs used in smaller SWTs have limitations such as relatively low voltage and high processing difficulty. Although EMGs generate considerable current, researchers are exploring the coupling of other energy conversion mechanisms to enhance the reliability of SWT.^189^^,^^190^

#### Piezoelectric effect

The PE effect results from the interaction between mechanical and electrical states in crystalline materials. There are two types of this effect: the direct PE effect and the inverse PE effect. In the direct PE effect, a PE material produces an electric potential when subjected to an external force. Conversely, the inverse PE effect creates internal mechanical strain when an electric field is applied to the material. The PE instanton equation is used to regulate both direct and inverse effects and is expressed as

(Equation 19) $$\left[\begin{array}{l} \delta \\ D \end{array}\right]=\left[\begin{array}{l} S^{E}\;d^{t}\\ d\;\varepsilon^{T} \end{array}\right]\left[\begin{array}{l} \sigma \\ E \end{array}\right]$$

where *σ* and *δ* are stress and strain components; *E* and *D* are electric field and a potential shift, respectively; *s, ε,* and *d* denote the elastic compliance, dielectric constant, and PE coefficient, respectively; the superscripts *E* and *T* denote the calculated constants t constant electric field and constant stress, respectively; and the superscript *T* denotes transposition.^191^

The PE generator (PEG) operates based on the direct PE effect, where PE materials deform and generate power in response to rectified mechanical energy.^192^ Conversion performance is determined by the material and structure, and more than 200 materials have shown potential for electromechanical conversion.^193^ Commonly used materials for SWTs include Pb(Zr,Ti)O3 (PZT), polyvinylidene fluoride (PVDF), quartz (SiO2), and various composites. PZT-based ceramics are polycrystalline with excellent performance, while PVDF can handle significant bending or deformation.^194^^,^^195^ In addition, researchers have developed polymer and nanocomposite-based piezoelectric structures to improve the properties of these materials. Classical structures such as cantilever beams,^196^ cymbals,^197^ and stacked arrangements^198^ (Figure 9B) are still popular. The cantilever beam is simple and easy to generate large strains, while the cymbal structure can withstand larger impact forces, and the stacked structure with multiple layers can withstand high mechanical loads. PEGs must operate at frequencies resonant with the device intrinsic frequency to run at maximum efficiency,^199^^,^^200^ so designers must select appropriate materials and structures based on the target application frequency and structure size.

#### Triboelectric effect

The TE effect occurs when two objects of different materials become charged after contact and separation.^201^ In many cases, the TE effect is perceived as a disadvantage, such as in oil tank transportation, textile workshops, or the interference of electronic components.^202^ In 2012, Wang’s research team proposed the triboelectric nanogenerator (TENG), a microscopic PGM that utilizes the TE effect, which has since garnered researchers' interest.^203^^,^^204^ By extending Maxwell’s displacement current formula, Wang^205^^,^^206^ provided the displacement current density of TENG as

(Equation 20) $$J_{D}=\varepsilon \frac{\partial E}{\partial t}+\frac{\partial P_{s}}{\partial t}$$

where *P*_*s*_ is the polarization due to the electrostatic surface charge caused by mechanical triggering.

TENG uses four main structures: contact separation (CS), lateral sliding (LS), single electrode mode (SE), and freestanding TE-layer (FT), shown in Figure 9C. In the CS structure, two triboelectric films of opposite polarity create areas of opposite charge, separated by an electrode.^207^ As the film vibrates from contact to separation due to an external force, electrons flow continuously between these electrodes, producing an AC output. The LS structure works in a similar way, but with one film moving relative to the other.^208^ SE, however, involves a mobile electrode in either CS or LS mode, producing an electrical output when contacted to the fixed electrode.^209^ Finally, FT uses a fixed symmetrical electrode and a free electrical layer; the difference in distance between the two fixed electrodes generates uneven charge distribution that causes electron flow and produces AC output.^210^ The structural versatility of TENG enables it to have excellent application flexibility, and many TENG-based rotating wind energy harvesters have been reported.^211^^,^^212^^,^^213^^,^^214^

#### Comparison and discussion

PGMs have distinct characteristics and are implemented in suitable SWTs with different structures and sizes. The attributes of each PGM determine the preferred mechanical energy excitation frequencies. For instance, EMGs are suitable for high-frequency conditions as the output voltage depends on the rate of flux change. In contrast, PEGs’ performance depends on material stress and strain, with a high reliance on intrinsic resonant frequency. TENGs operate best under low-frequency conditions because of their limited friction characteristics. Excitation frequencies also reveal the appropriate rotor type for PGMs. Horizontal and Darrieus rotors are suitable for EMGs to effectively utilize wind energy in high-wind environments, whereas Savonius rotors or wind cups pair well with TENGs for harnessing breeze energy. PEGs require structure and rotor adaptation to operate at resonant frequencies and are more suitable for use in stable wind fields. While EMGs are typically on a larger scale, which allows for more turns or larger-wire-diameter coils, size reduction increases the prevalence of simpler structures and higher power density of PEGs and TENGs, making them preferred PGMs. Understanding the electrical output characteristics of PGMs is vital for practical applications. EMGs have low internal resistance, resulting in low voltage and high current output, while the opposite is accurate for PEGs and TENGs.

In conclusion, PGMs' design is influenced by the operating principle, excitation characteristics, coupling rotor, and environment. Table 2 summarizes and compares various PGM types' principles, excitation frequencies, main features, output characteristics, and applicable rotors, providing guidance for designing SWT-oriented PGMs.

**Table 2:** Comparative analysis for different PGMs

Types	Principle	Structures	Characteristics	Limitations	Applicability
EMG	Flux changes with magnet-coil motion	• Radial flux • Axial flux	• Low voltage & high current • High output capability • High-frequency response	• Complex structure • Poor performance at micro size • Inefficient at low frequencies	• Macro size • High wind speed • Horizontal or Darrieus rotor
PEG	Deformation forces stress/strain in PE material	• Cantilever beam • Flexural structure • Stacked structure	• Medium voltage & current • Simple and flexible structure • Resonant frequency response	• Hard to couple rotors • Limited operating frequency	• Unlimited size • Stable wind speed • Depends on construction
TENG	Triboelectric films produce potential differences post-separation	• Contact separation • Lateral sliding • Single electrode • Freestanding TE-layer	• High voltage & low current • High power density • Simple and flexible structure	• Limited operating life generation potential	• Micro size • Low wind speed • Savonius rotor or wind cup

### State-of-the-art research trends

#### Electromagnetic generator

**Figure 10:**
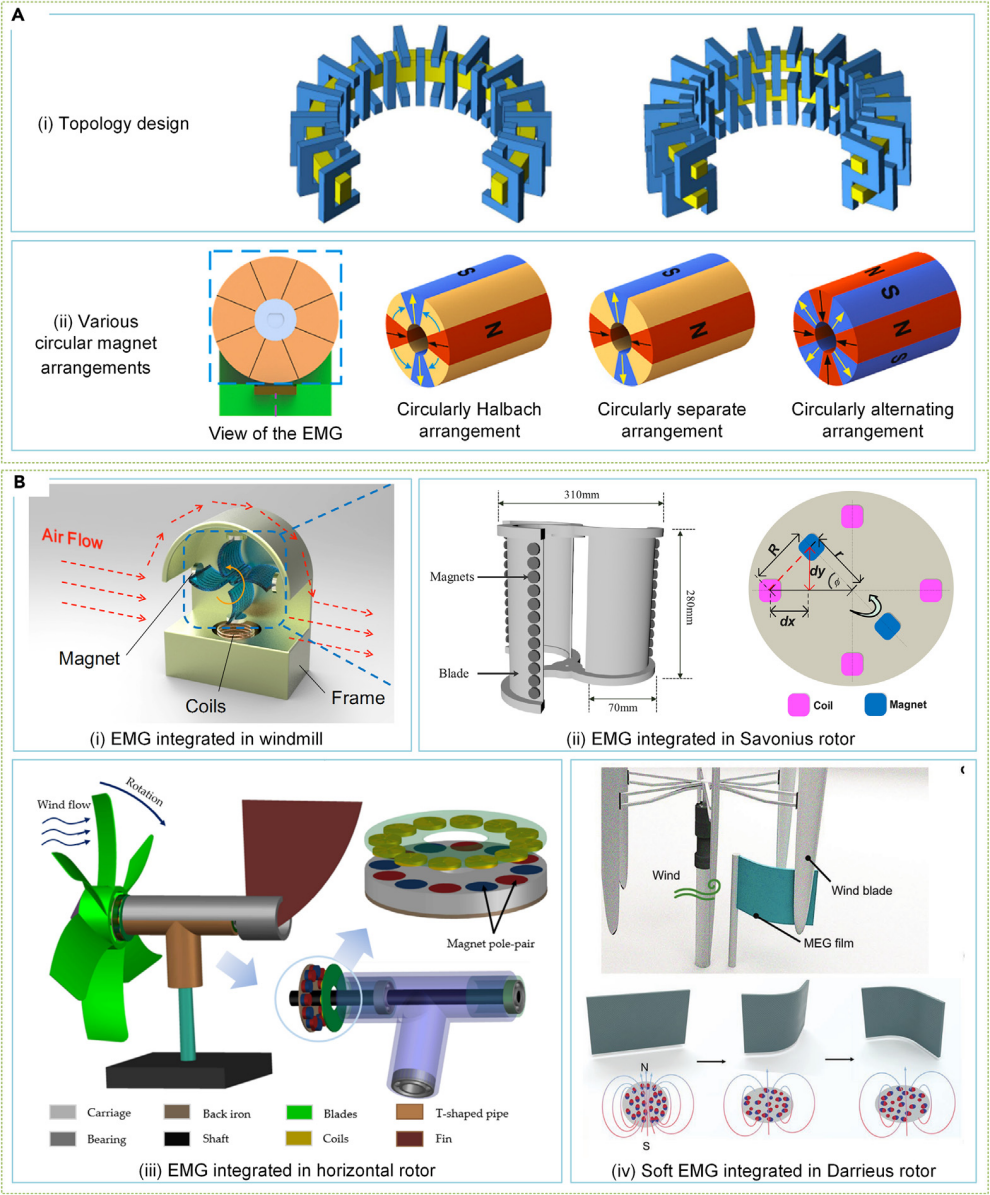
Design and optimization of small EMGs for SWT (A) Small EMG design and analysis.^219^^,^^222^ (Sharif University of Technology Publishing.^219^ Elsevier Publishing.^222^ Reproduced with permission. All rights reserved). (B) Integrating EMGs with rotors.^225^^,^^226^^,^^228^^,^^229^ (Elsevier Publishing.^225^^,^^226^ MDPI Publishing.^228^ Wiley Publishing.^229^ Reproduced with permission. All rights reserved).

The performance limitations of EMGs due to ruler shrinkage are critical in SWTs.^215^ Researchers have been primarily focused on small EMG design^216^^,^^217^^,^^218^ to overcome this issue. Ajamloo et al.'s axial flux PMG structure for SWTs avoids even-order resonance and balances the voltage waveform by using skewed teeth to reduce cogging torque, as displayed in Figure 10Ai.^219^ Huang et al.^220^ analyzed superconducting PMGs' electromagnetic performance with iron-core and air-core stators and found that the iron-core stator has better torque capability at the expense of greater torque pulsation. Ram Kumar and Latha^221^ studied the pole number for small switched reluctance generators for SWT orientation and determined that 8/6 and 12/8 configurations perform better. Li et al.^222^ analyzed the circular magnet arrangements' effect on micro-EMGs' output performance and found that EMGs with alternating magnet configuration had the most satisfactory charging performance in micro-capacitors (Figure 10Aii). Despite EMGs' limited performance in low-frequency excitation or size limitations, micro-EMGs are increasingly being used as PGMs for SWTs due to topology optimization and micro and nano-fabrication technology.^223^^,^^224^

Integrating the EMG with the SWT rotor can solve the size limitation problem of EMG performance due to the similarity between their topologies. Wu and Lee^225^ proposed a miniature windmill energy harvester based on the EM effect shown in the figure, where the magnet fixed on the rotating blade acts as the rotor of the EMG, and the coil set on the base comprises the stator, as visible in Figure 10Bi. Tairab et al.^226^ increased the EMG’s structural space by placing the magnet at the blade’s edge and fixing the coil on the outer deflector (Figure 10Bii), and a similar design also appears in the work of Fang et al.^227^ Roy et al.^228^ integrated an axial flux EMG inside a horizontal rotor to improve the output while harvesting wind energy, as illustrated in Figure 10Biii. In addition, Zhao et al.^229^ designed a soft magnetoelastic generator that uses the magnetoelastic effect and electromagnetic induction, as presented in Figure 10Biv. This EMG generates power as the rotor blades periodically tap a soft magnet made of a soft magnetic polymer and metal coil, providing another EMG design approach.

#### Piezoelectric generator

**Figure 11:**
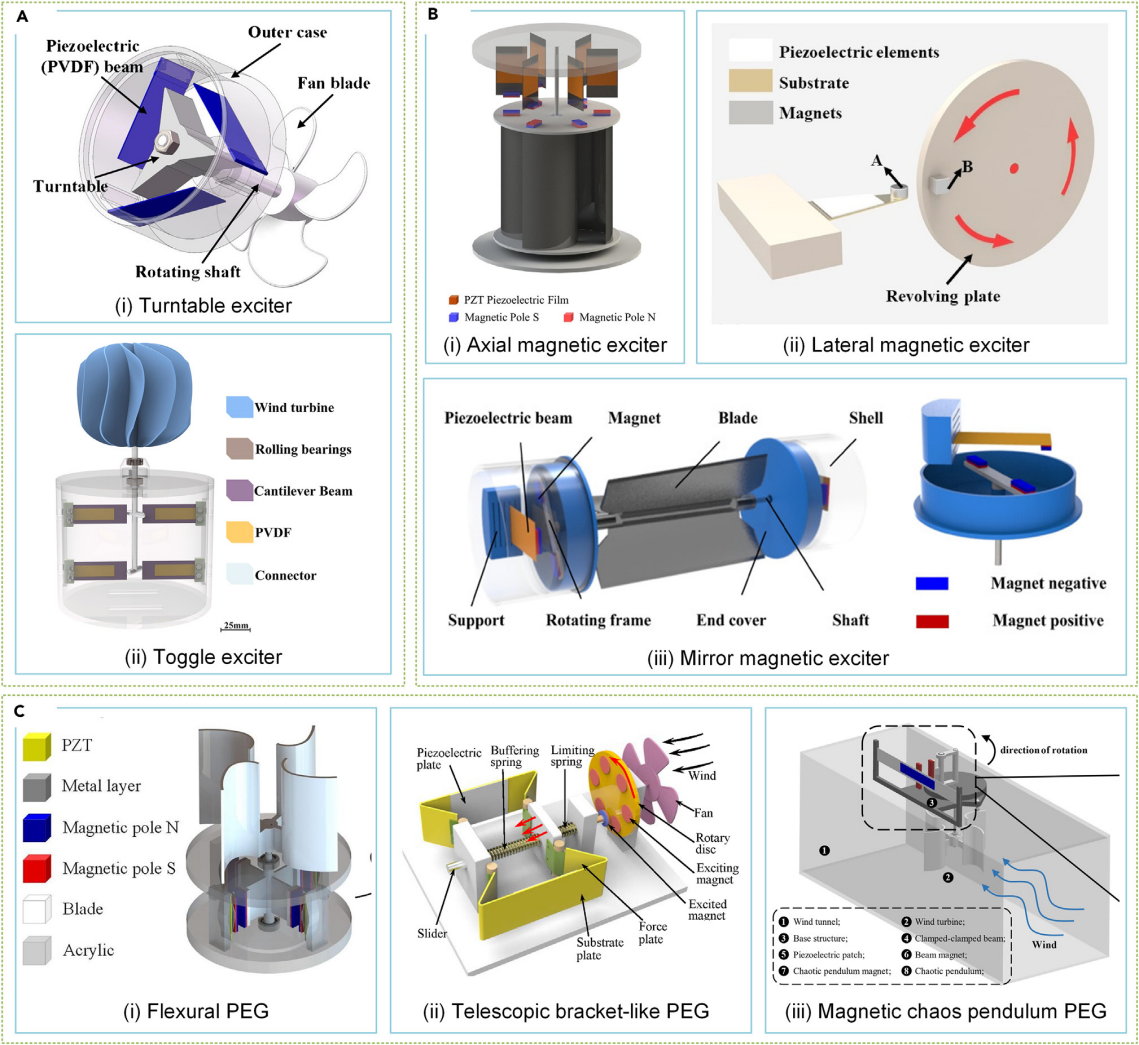
Various PEGs for SWT (A) Mechanical cantilever PEGs.^234^^,^^235^ (Elsevier Publishing.^234^^,^^235^ Reproduced with permission. All rights reserved). (B) Magnetic cantilever PEGs.^232^^,^^238^^,^^242^ (Elsevier Publishing.^232^^,^^238^^,^^242^ Reproduced with permission. All rights reserved). (C) Non-cantilever PEGs.^243^^,^^245^^,^^246^(Elsevier Publishing.^243^^,^^245^^,^^246^ Reproduced with permission. All rights reserved).

PEGs have been developed for SWTs, necessitating additional exciters due to their non-rotating topology that is not conducive to direct rotor coupling.^230^^,^^231^ Cantilever beam structures are ideal for excitation due to their large deformation and wide frequency response. Mechanical or magnetic excitation are the two common forms available for cantilever beam exciters.^232^^,^^233^ The former involves periodic impact for deformation of the piezoelectric sheet during rotation, while the latter employs magnetic poles for force. He et al.^234^ designed an SWT using mechanical-excited PEGs, determining optimal speed by analyzing intrinsic frequency for a power output of 36.89 mW at 16 m/s wind speed (Figure 11Aii). Zhang et al.^235^ suggested improving power generation performance using a method to adjust the vibration frequency of the piezoelectric sheet for another mechanical-excited PEG, as shown in Figure 11Ai.

Magnetic excitation is preferred over mechanical excitation since the former can eliminate static torque, improve starting performance, and increase the lifetime of piezoelectric sheets. Karami et al.^236^ proposed a magnetic-excited PEG using magnets with opposite magnetism to oscillate the piezoelectric sheet, inspiring similar designs by other researchers, as displayed in Figure 11Bi.^237^^,^^238^^,^^239^ Wang et al.^232^ developed and analyzed the impact of piezoelectric sheet shape on the electrical properties of their PEG, as depicted in Figure 11Bii. Similar structures were employed in studies conducted by others.^240^^,^^241^ Yu et al.^242^ proposed and analyzed in great detail the mirror-rotation PEG’s effect of parameters like magnet distance on output performance, visualized in Figure 11Biii.

Remarkable PEGs have been proposed for SWT in addition to cantilevers. Zhao et al.^243^ created a flexural PEG (Figure 11Ci) that uniformly amplifies magnetic repulsion to apply it to the piezoelectric ceramic effectively, preventing nonlinear resonance issues. Tao et al.^244^ achieved 150W power output from a 1-m-diameter rotor by using a Scotch yoke to convert rotation into piezoelectric sheet vibration. Kan et al.^245^ devised a telescopic bracket-like PEG to prevent excessive cantilever deformation in extreme input conditions, demonstrated in Figure 11Cii. Chen et al.^246^ introduced a PEG with a chaotic magnetic pendulum that generates chaotic beam vibration continuously and stably, even under unpredictable natural wind conditions, as displayed in Figure 11Ciii.

#### Triboelectric nanogenerator

**Figure 12:**
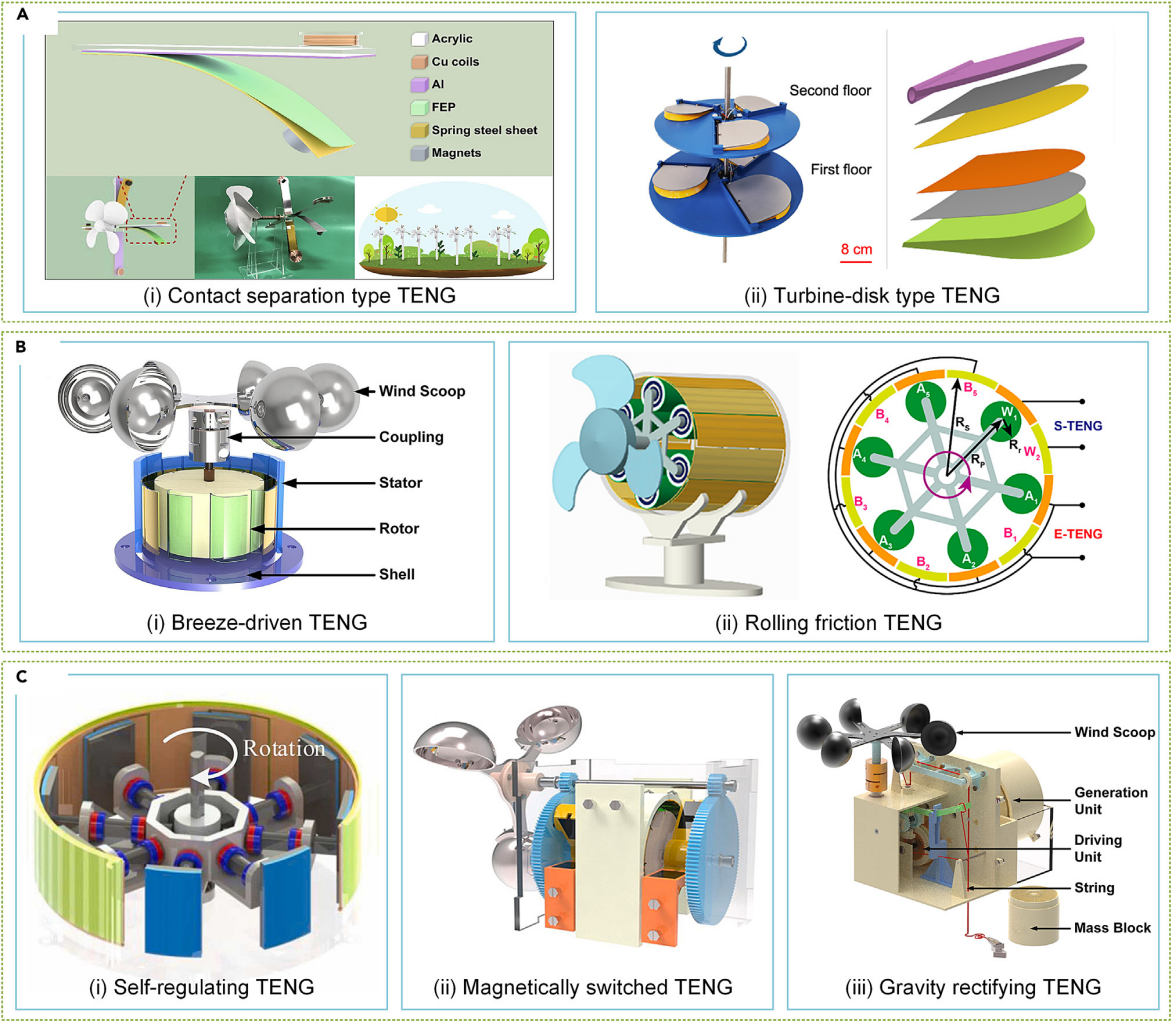
Various TENGs for SWT (A) Contact separation TENGs.^247^^,^^248^ (Springer Publishing.^247^ Elsevier Publishing.^248^ Reproduced with permission. All rights reserved). (B) Sliding friction TENGs.^213^^,^^249^ (Elsevier Publishing.^213^ ACS Publishing.^249^ Reproduced with permission. All rights reserved). (C) Structural design for TENGs.^250^^,^^251^^,^^252^ (Elsevier Publishing.^250^^,^^251^^,^^252^ Reproduced with permission. All rights reserved).

A range of basic structures has potential for TENGs in SWT applications. Zhang et al.^247^ proposed the contact separation TENG windmill, which uses cantilevered spring steel plates as electrodes, driven by periodic contact separation due to rotational coupling with gravity (Figure 12Ai). Meanwhile, Gao et al.^248^ developed a turbine-disk TENG that undergoes periodic contact separation and minimizes wear under rotating conditions (Figure 12Aii). Sliding friction TENGs require less motion space, similar in topology to EMG. For instance, Li et al.^213^ proposed a radial layout breeze-driven TENG that can attain a maximum power of 2.81 mW through sliding friction between the FEP film and copper electrode, driven by wind cups. Unfortunately, sliding friction can cause increased wind resistance and reduced lifetime (Figure 12Bi). To alleviate this, Fu et al.^249^ proposed a planetary frame structure rolling friction TENG that operates at a wind speed of 2 m/s and performs well in windy environments (Figure 12Bii).

To improve the comprehensive performance of TENG-based SWTs, researchers have proposed a number of enhancements. As shown in Figure 12Ci, Zhou et al.^250^ introduced a self-regulating strategy that uses centrifugal and magnetic forces to reduce damage from sliding friction, resulting in minimal frictional resistance at low wind speeds and increased contact at high wind speeds. Yong et al.^45^ designed an auto-switching dual-rotor TENG that compensates for the advantages of two wind cups in different wind speed zones, thereby enhancing the output performance of SWTs. Stable output requires a continuous and stable input for the TENG, which presents a challenge when working with the intermittent natural wind. To address this, Liu et al.^251^ proposed a magnetically switched TENG (Figure 12Cii) that generates electrical energy steadily at variable and irregular wind speeds. Finally, Wang et al.^252^ suggested converting wind energy into gravitational potential energy and subsequently into stable electrical energy (Figure 12Ciii).

#### Hybrid integrated generators

**Figure 13:**
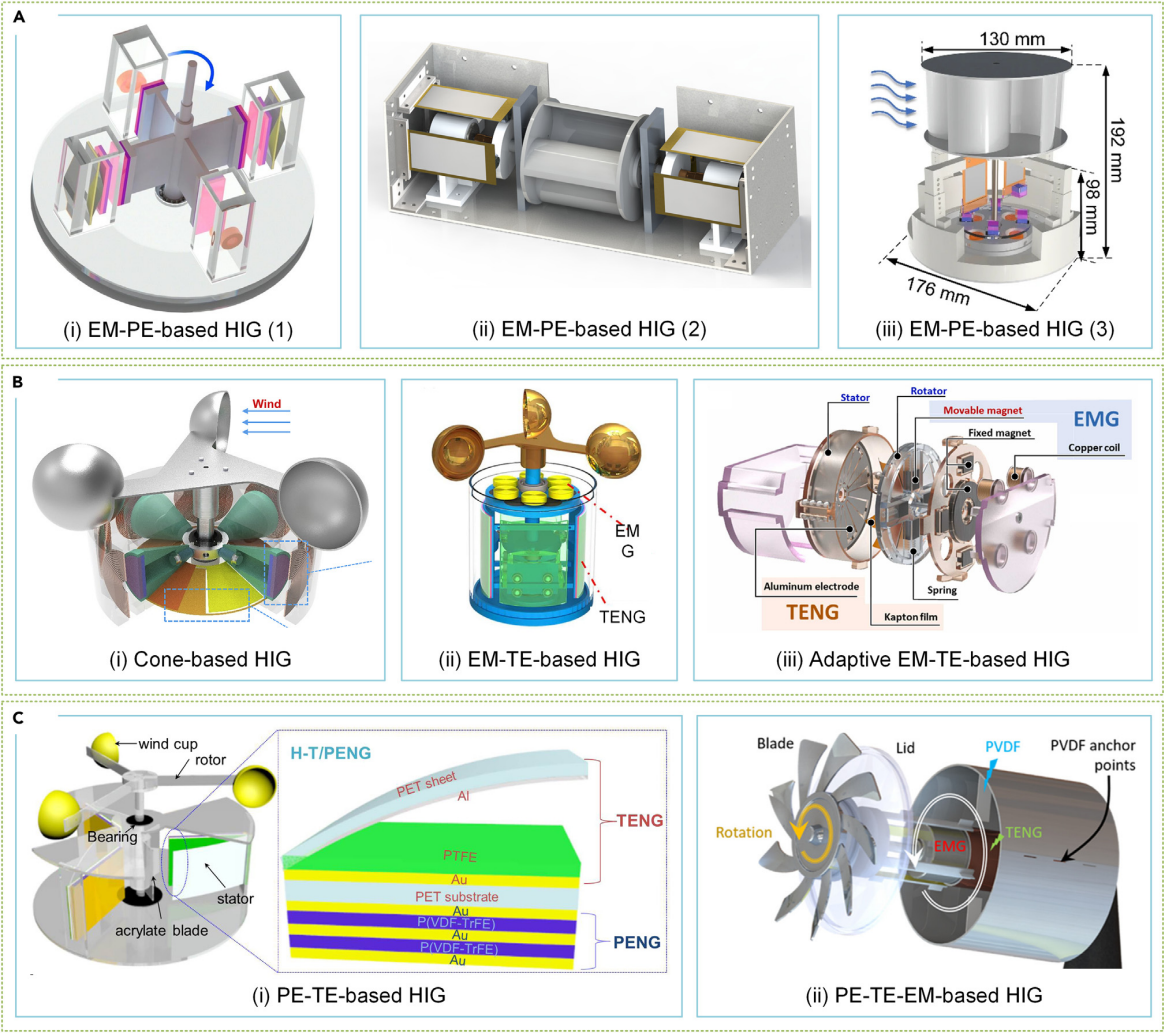
Various HIGs for SWT (A) EM-PE-based HIGs.^253^^,^^254^^,^^255^ (Elsevier Publishing.^253^^,^^254^ Springer Publishing.^255^ Reproduced with permission. All rights reserved.). (B) EM-TE-based HIGs.^190^^,^^256^^,^^257^ (Elsevier Publishing.^190^^,^^256^^,^^257^ Reproduced with permission. All rights reserved.). (C) PE-TE-based HIGs.^185^^,^^258^ (Elsevier Publishing.^185^^,^^258^ Reproduced with permission. All rights reserved).

Combining multiple PGMs in an effective manner can significantly enhance the efficiency of space utilization and power density of SWTs, leading to more stable and reliable performance output as well as an expanded operating range. Many hybrid integrated generators (HIGs) have been developed for SWTs, leveraging their advantageous properties. Researchers integrate EMG with PEG or TENG to form HIGs, improving output performance due to the high current output of EMG. Zhao et al.^253^ proposed an EM-PE-based HIG for wind energy harvesting that features symmetrically opposite magnetic arrangements, minimizing resistance torque and improving flux variation, leading to high power density and mechanical durability, as illustrated in Figure 13Ai. Meanwhile, Zheng et al.^254^ developed an EM-PE-based HIG for subway tunnels (Figure 13Aii), utilizing micro bearings to reduce friction between the cam and the piezoelectric sheet. Cao et al.^255^ improved the output power of EMG by adding a conductive slip ring based on a conventional PEG, as presented in Figure 13Aiii.

Like the EM-PE-based HIG, the integration of TE and EM generators can also produce significant output current and voltage simultaneously. Fang et al.^256^ obtained a power density of 72.1 W/m^3^ by using a tapered roller as the rotor of the EMG and a separate layer of the TENG, as illustrated in Figure 13Bi. Mu et al.^257^ designed an EM-TE-based HIG to operate in broadband wind speeds by considering the different operating frequencies of EMG and TENG, as shown in Figure 13Bii. For greater flexibility in utilizing the advantages of EMG and TENG, Li et al.^190^ developed an adaptive HIG based on moving magnets, which switches the power generation form according to the input wind speed adaptively, as indicated in Figure 13Biii. Moreover, the integration of PE-TE or PE-TE-EM forms some promising designs owing to the similar output characteristics and mechanical response of PE and TE. As visible in Figure 13Ci, Zhao et al.^258^ developed a high-performance PE-TE-based HIG that effectively produces stable energy output by activating the PEG resonant mode and minimizing output phase difference. Egbe et al.^185^ proposed a PE-TE-EM HIG (Figure 13Cii) that considerably enhances space utilization and system output at low frequency wind speeds.

## Sustainable IoT based on SWTs

The rapid advancement of IoT has established an extensive network of low-power devices such as sensors, controllers, communicators, and memories.^259^^,^^260^ Based on a report by the International Data Corporation, the number of connected IoT devices is projected to reach 55.7 billion by 2025. The extensive exchange of information among these devices creates a significant energy demand. In response, the idea of a sustainable IoT system has emerged, with a focus on utilizing SWTs as the energy source for IoT devices, in order to align with carbon neutrality objectives. To sustainably and inexpensively power IoT applications, SWT technology aligns well with their low-power and widespread energy requirements, as wind energy is almost all-pervasive.^261^^,^^262^ Utilizing SWTs technology, sustainable IoT systems can harness ambient energy to achieve a consistent and long-lasting power supply. This approach significantly minimizes the need for frequent maintenance interventions and reduces associated costs. The most efficient WER and PGM for IoT depend on the application scenarios, including transportation,^263^ urban environments,^264^ intelligent agriculture,^265^ and self-powered wind sensing.^266^ An overview of SWT applications based on these scenarios showcases their characteristics, latest advancements, challenges, and potential opportunities for IoT.

### Transportation

Transportation is a crucial element for social and economic growth and accounts for a significant portion of energy consumption.^267^ The global transportation network incorporates various IoT applications essential for ensuring seamless transportation,^268^ a primary objective of SWT. Land, marine, and air transportation classify transportation modes. However, as the latter two lack the infrastructure for SWT installation, research into SWT for transportation has mainly concentrated on land transportation. The shift to electric-powered land transportation has resulted in increased energy consumption. The extensive deployment of electrical equipment along roadways currently relies on either non-eco-friendly, disposable electrochemical batteries or expensive wired power supply networks. Incorporating distributed SWTs could be an effective solution to this issue. A comprehensive review of past and current research on the application of SWT in transportation is necessary.

**Figure 14:**
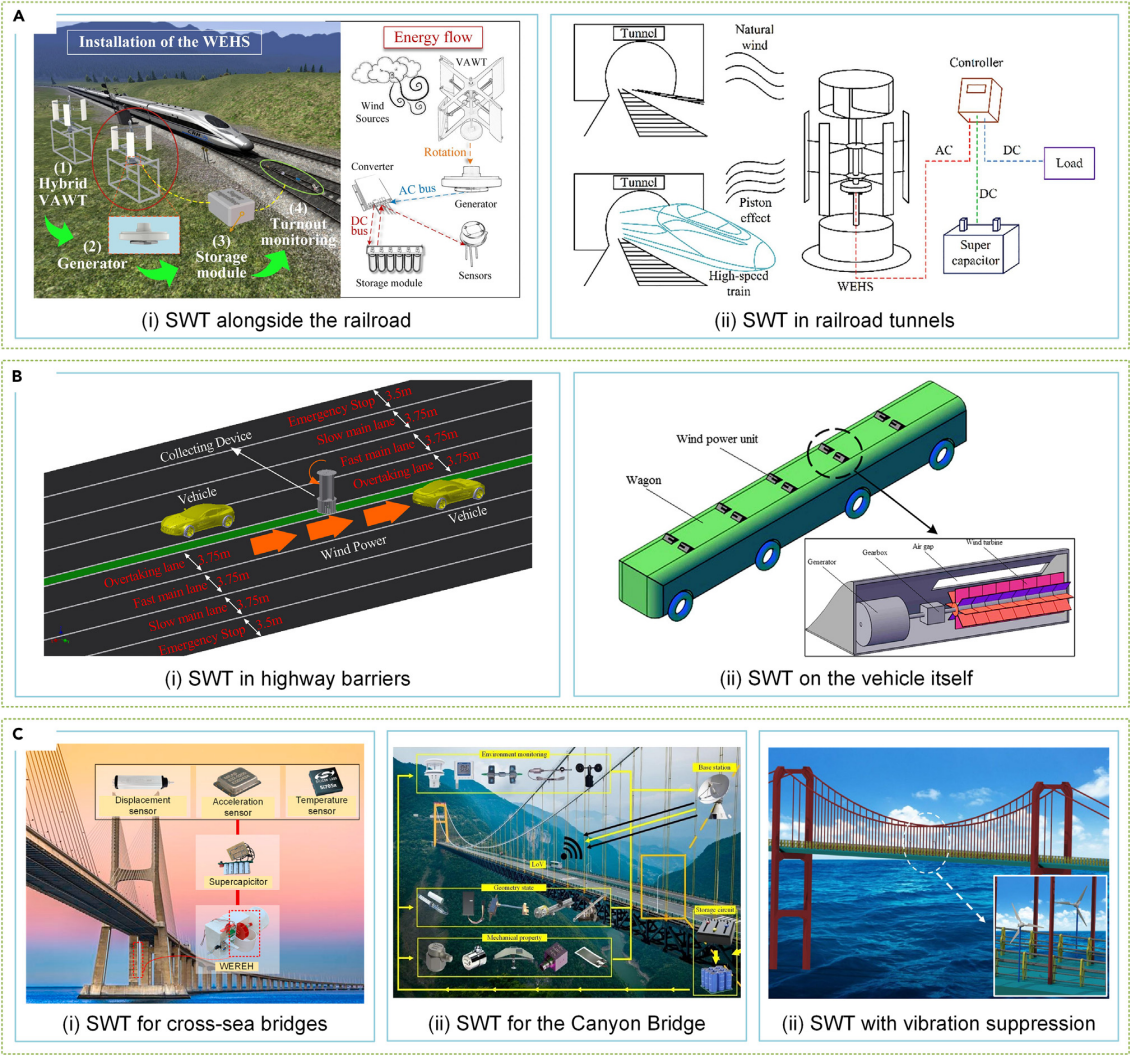
SWTs in Transportation (A) SWTs for railroad applications.^271^^,^^272^ (Elsevier Publishing.^271^^,^^272^ Reproduced with permission. All rights reserved.). (B) SWTs for highway applications.^279^^,^^282^ (Elsevier Publishing.^279^ Springer Publishing.^282^ Reproduced with permission. All rights reserved.). (C) SWTs for large-span bridges.^25^^,^^287^^,^^288^(Elsevier Publishing.^25^^,^^287^^,^^288^ Reproduced with permission. All rights reserved).

Several studies have explored SWT in railway transportation.^9^^,^^269^ Given the vast and interconnected railroad network, it is challenging to identify an environmentally friendly energy source that can provide coverage, except for the omnipresent wind energy. Researchers have analyzed wind energy generated by fast-moving trains. Zhang et al.^270^ created an elastic TENG set, situated near railway tracks, that reduces friction and wear while enhancing durability four times by decreasing rotational torque. This solution shows potential as an energy supply for future high-speed railway systems. Similarly, Wang et al.^271^ proposed an adaptive pitch VAWT that leverages abundant wind resources to power high-speed rail equipment on the Tibetan plateau, as shown in Figure 14Ai. Pan et al.^26^ incorporated SWT into a wind barrier set that harnesses wind energy and reduces the impact of strong winds on trains in high wind regions. The piston effect in tunnels generates significant wind energy that’s worth harnessing. Pan et al.^272^ developed a hybrid VAWT that combines piston wind energy and natural wind energy harvesting with Savonius and H-Darrieus rotors, as illustrated in Figure 14Aii. Several similar designs have been reported in studies.^254^^,^^273^ After analyzing various SWT performance metrics in tunnels, Guo et al.^274^ predicted that the 4.8 × 10^12^ J wind energy potential, based on China’s tunnel distribution, could meet the electricity demand of distributed equipment in tunnels.

Wind energy’s potential in road transport has also attracted attention from researchers, especially for highways. Hegde et al.^275^ investigated the viability of installing SWTs on highways. Han et al.^276^ estimated the unit energy that could be harvested from various vehicle models using SWTs designed for highways, as discussed in studies.^277^^,^^278^ Figures 14Bi shows Hu et al.'s experiments on the use of SWTs on highways.^279^^,^^280^^,^^281^ They first optimized SWTs to harvest convective wind energy using cars and trucks as a source of wind.^279^ Next, they proposed a dust collection device powered by convective wind energy designed for highway application^280^ and analyzed SWT performance with different lanes, obtaining a maximum power factor of 28.2%.^281^ Some researchers have also studied SWTs on vehicles. As shown in Figure 14Bii, Nurmanova et al.^282^ investigated the feasibility of implementing SWTs on moving vehicles, finding that the power generated is sufficient to offset air resistance and support the load. Khan et al.^283^ proposed using SWTs to support communication devices in electric vehicles, identifying optimal locations and generating direction. Zhao et al.^284^ developed adjustable SWTs, installed close to the vehicle’s front grille, which directly powered low-energy devices on the vehicle, thereby demonstrating significant performance retention.

Large-span bridges are essential transportation facilities for navigating difficult terrain. Their locations, such as canyon and sea-crossing bridges, are often subjected to significant wind.^285^^,^^286^ Despite the challenges posed by the complex wind environment, these areas provide a notable wind energy resource that has attracted the attention of researchers. Cao et al.^287^ designed a hybrid energy harvester (Figure 14Ci), installed on a cross-sea bridge pipeline, that used Savonius rotors to collect wind energy. They subsequently proposed an EM-PE hybrid wind energy harvester that achieved a maximum power output of 19.24 mW.^255^ Tan et al.^25^ developed a mechanical wind speed adaptive module SWT to study the coupling effect of the canyon wind field and obtain the wind speed characteristics around the canyon bridge, as presented in Figure 14Cii. Furthermore, Zhang et al.^288^ successfully harnessed the vorticity variation of airflow through the horizontal-axis rotor to convert wind energy into electrical energy, effectively minimizing vortex-induced vibration on the bridge (Figure 14Ciii).

### Urban environment

The global increase in urbanization and industrialization has led to a greater demand for energy, especially in developing nations. Building complexes, especially those found in cities, provide ideal deflection surfaces, accelerating airflow and presenting promising avenues for energy harvesting.^289^^,^^290^ By deploying SWTs in urban areas alongside IoT technologies, we can help alleviate stress on the primary grid while also promoting sustainable city development.

**Figure 15:**
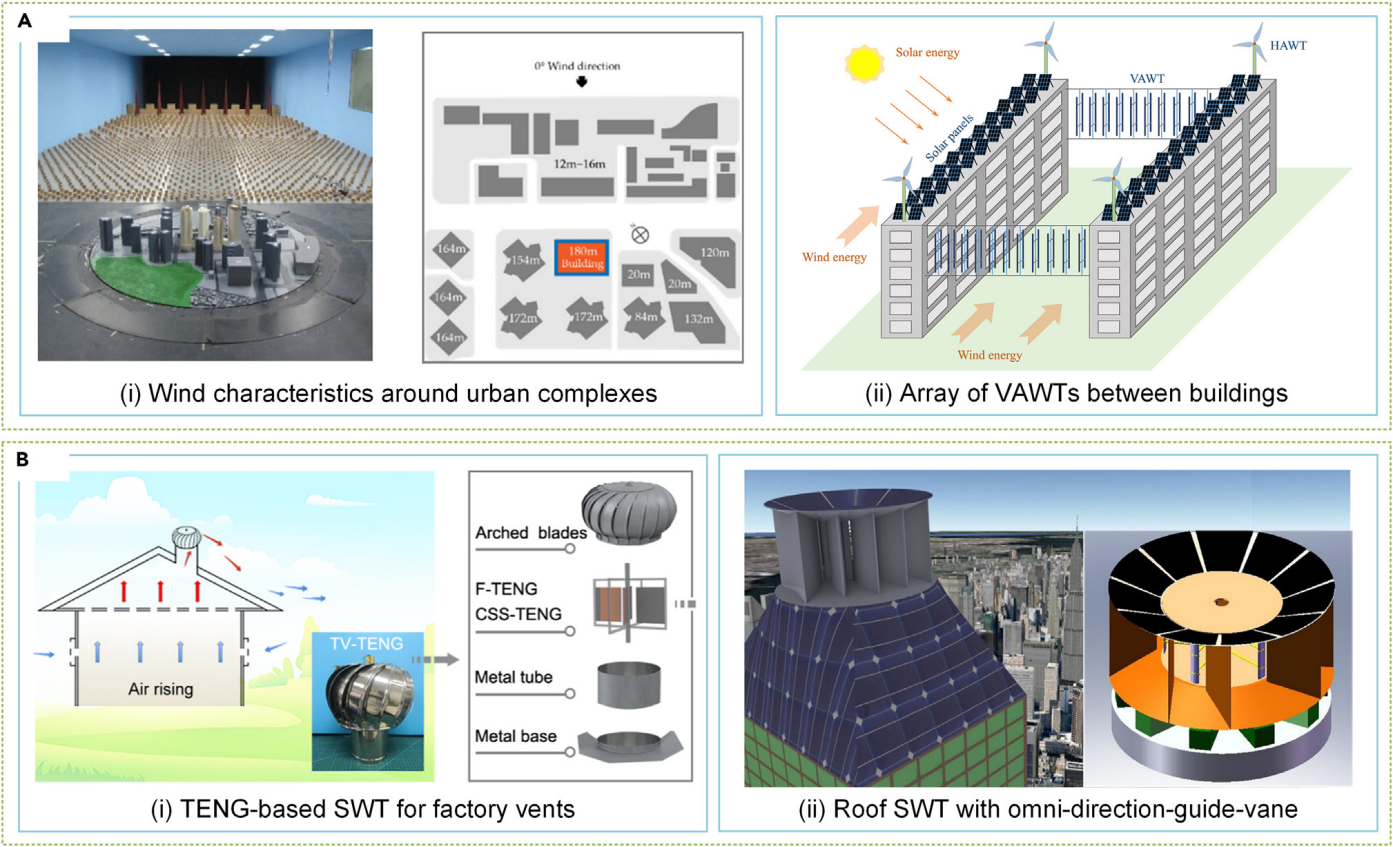
SWTs in urban environments (A) The gaining effect of buildings on SWT.^292^^,^^296^(MDPI Publishing.^292^ Elsevier Publishing.^296^ Reproduced with permission. All rights reserved.). (B) SWTs for the urban environment with low noise and high safety.^297^^,^^300^(ACS Publishing.^297^ Elsevier Publishing.^300^ Reproduced with permission. All rights reserved).

Generating power through SWTs in complex urban environments requires an examination of their characteristics. Hemida et al.^291^ conducted wind tunnel experiments to measure wind speed and pressure distribution above a high-rise building’s roof. Park et al.^292^ studied the wind pressure distribution around the same building, as demonstrated in Figure 15Ai. Moreover, Ruiz et al.^293^ delved into the aerodynamics of circular penetrations, used as SWT diffusers, in high-rise buildings, while Hassanli et al.^294^ explored other penetration shapes. VAWTs could be installed on building sides or between two buildings, according to Xu et al.,^295^^,^^296^ equipped with deflector components acting as diffusers, shown in Figure 15Aii.

However, using high-turbulence wind energy to generate power in urban areas through SWTs is a challenging research topic. Efforts have been made in this area, with researchers such as Zhang et al.,^297^ who demonstrated a vent TENG-based SWT for industrial production safety monitoring in an industrial plant, as depicted in Figure 15Bi. Li et al.^298^ studied the performance of a dual VAWT in an urban setting and emphasized the need to consider skewed flow performance degradation when designing the VAWT layout. In addition, Zhang et al.^299^ analyzed the flow characteristics of SWT units installed on building complex roofs and found that the relative power of SWTs increases and decreases with the rotor’s height from the roof. However, using SWTs in urban areas raises concerns about noise, light, shadow pollution, and safety issues associated with rotating blades. To address these issues, Chong et al.^300^ developed an all-guide blade device that improves SWT performance while minimizing safety and negative visual impacts in urban areas, as illustrated in Figure 15Bii. Nardecchia et al.^301^ designed and optimized a ducted SWT for urban settings, improving the rotor’s omnidirectionality and minimizing environmental damage caused by exposed rotors.

### Intelligent agriculture

**Figure 16:**
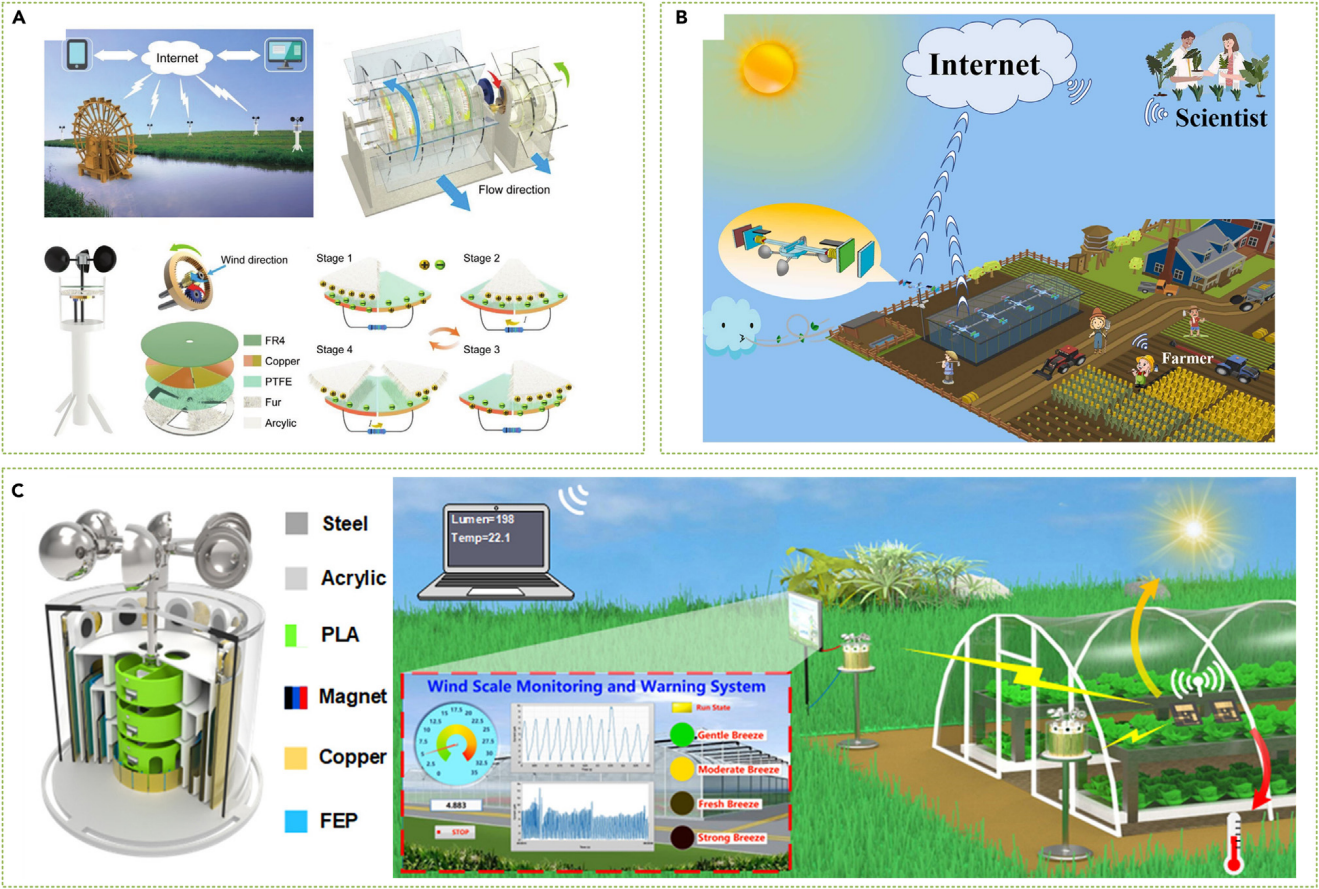
SWTs in intelligent agriculture (A) Moisture-tolerant SWT for intelligent agricultural environments.^304^(Wiley Publishing.^304^ Reproduced with permission. All rights reserved.). (B) SWT for agriculture with coupled solar cells.^307^ (Elsevier Publishing.^307^ Reproduced with permission. All rights reserved.). (C) SWT with wind level warning and power regulation.^306^(Elsevier Publishing.^306^ Reproduced with permission. All rights reserved).

The IoT’s rapid growth has led to the rise of agricultural information intelligence, as IoT-backed applications have made it possible to monitor and control crop environments in real time.^302^^,^^303^ In this regard, the deployment of SWTs to power sensors throughout farmland is crucial to the development of agricultural intelligence. As presented in Figure 16A, Chen et al.^304^ designed a unique TENG-based SWT made of animal fur with low sensitivity to environmental humidity, which could serve as a potential smart agriculture solution. Moreover, a breezy environment promotes crop health. To harness natural breeze energy, Li et al.^213^ created a breeze-driven SWT using wind cups and TENG, which generated a noteworthy 2.81 mW of power output at a 4-m/s wind speed. Gui et al.^305^ proposed a hybrid EM-TENG SWT with a stacked structure that performed exceptionally between wind speeds of 3 m/s and 6 m/s. Dang et al.^306^ improved the wind energy harvesting capacity of their EM-TENG-based SWT using power dynamic matching and wind warning mechanisms to avert damage to crops and agricultural facilities due to high winds, as displayed in Figure 16C. Wang et al.^307^ combined commercial solar cells with SWTs to create a self-powered temperature and humidity monitoring system for intelligent agriculture, demonstrated in Figure 16B.

### Self-powered wind sensing

**Figure 17:**
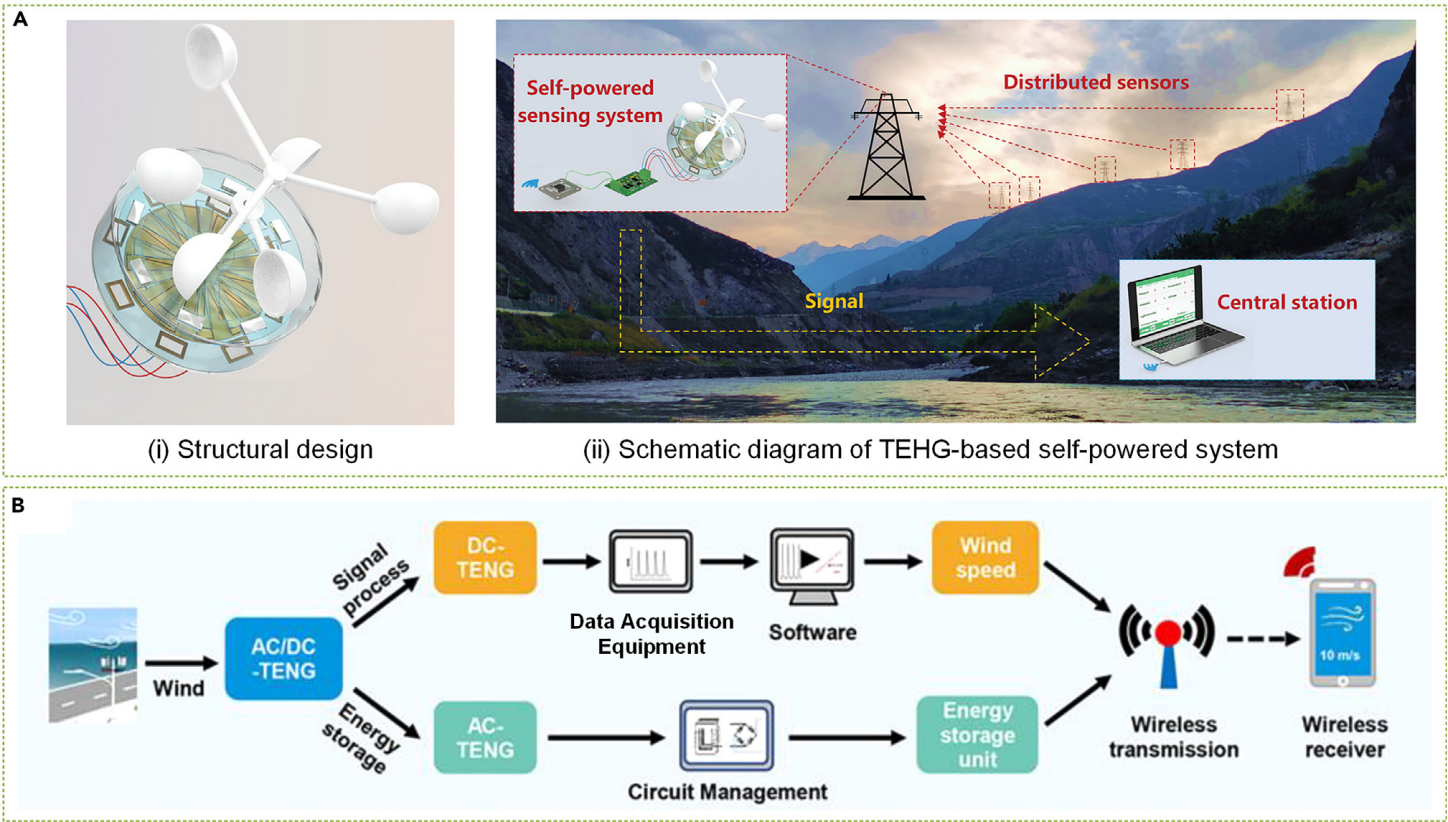
SWTs for self-powered wind sensing (A) Triboelectric-electromagnetic hybridized nanogenerator (TEHG) based self-powered system.^314^ (Wiley Publishing.^314^ Reproduced with permission. All rights reserved.). (B) Work flowchart of the self-powered wind monitoring system.^316^(ACS Publishing.^316^ Reproduced with permission. All rights reserved).

Sensing systems, particularly wind technology, serve as an essential component of the IoT, integral to transportation, city planning, and agriculture. Self-powered SWTs can carry out real-time wind speed monitoring in addition to harnessing wind energy for conventional wind sensors.^308^^,^^309^ Sardini and Serpelloni^310^ introduced the EMG-based self-powered wind sensor for pipeline wind monitoring, which was later utilized for remote sensing of wind-driven wildfires.^311^ TENG technology is proving to be effective in real-time wind speed monitoring by analyzing its output voltage frequency. Wang et al.^312^ demonstrated the feasibility of TENG as a self-powered wind sensor with a hybrid SWT for temperature and humidity sensors. Later studies by Fan et al.^313^ and other researchers^314^^,^^315^^,^^316^^,^^317^ further refined the concept of self-powered wind sensing through SWTs. Among these studies, Yong et al.^314^ established a dual-channel power management topology to enhance SWTs' environmental adaptability and stability (Figure 17A). Zhao et al.^315^ proposed a bionic SWT based on a callus structure for wind direction and speed detection, and He et al.^316^ demonstrated a strategy for fully self-powered and real-time wind speed monitoring, as displayed in Figure 17B.

### Summary of IoT applications

IoT-based wind energy systems offer a multitude of opportunities to engineer SWTs with varying PGMs, WER, and structures. These may be customized to suit applications for wind energy harvesting or self-operating wind sensing across different environments. Table 3 highlights an overview of developed SWTs for distributed wind energy harvesting categorized by their WER, PGM, and performance.

**Table 3:** The development of SWT for distributed wind energy harvesting on WER, PGM, and performance

Reference	Year	WER	PGM/Simulation	Rotor diameter	Wind Speed	Power	Application
Bethi et al.^273^	2019	Savonius	CFD	0.75 m	18 m/s	738.92 W	Rail tunnel
Wang et al.^271^	2021	Darrieus	EMG	0.52 m	13 m/s	1.08 W	Rail-side
Zheng et al.^254^	2021	Savonius	EMG & PEG	80 mm	7 m/s	59.31 mW	Subway tunnel
Pan et al.^26^	2022	Horizontal	EMG	90 mm	12 m/s	379.3 mW	Rail wind barrier
Pan et al.^272^	2019	Hybrid	EMG	250 mm	11 m/s	107.76 mW	Rail tunnel
Zhang et al.^270^	2021	Darrieus	TENG	320 mm	600 rpm	9.5 mW	Rail tunnel
Bani-Hani et al.^277^	2018	Darrieus	EMG	1,800 mm	5 m/s	50 W	Highway side
Hu et al.^280^	2023	Savonius	CFD	1 m	–	–	Highway barrier
Khan et al.^283^	2021	Horizontal	EMG	0.36 m	25 m/s	100 W	Car front
Zhao et al.^284^	2022	Savonius	MATLAB	120 mm	12 m/s	7.1 W	Car front
Cao et al.^287^	2021	Savonius	EMG & PEG	136 mm	12 m/s	2.19 mW	Cross-sea bridge
Cao et al.^255^	2022	Savonius	EMG & PEG	130 mm	6.5 m/s	19.24 mW	Canyon bridge
Tan et al.^25^	2022	Darrieus	EMG	242 mm	14 m/s	169.0 mW	Canyon bridge
Xu et al.^296^	2021	Darrieus	CFD	1.7 m	–	–	Between buildings
Zhang et al.^297^	2021	Savonius	TENG	∼290 mm	7 m/s	2.71 mW	Factory vents
Chong et al.^300^	2013	Darrieus	EMG	1.22 m	6 m/s	0.4352 W	Roofs
Park et al.^330^	2015	Savonius	EMG	0.3 m	16 m/s	∼310 W	Residential buildings
Chen et al.^304^	2021	Wind cup	TENG	∼0.3 m	23 m/s	56.79 mW	Intelligent agriculture
Li et al.^213^	2022	Wind cup	TENG	∼0.3 m	4 m/s	2.81 mW	Intelligent agriculture
Gui et al.^305^	2022	Wind cup	EMG & TENG	120 mm	6 m/s	118.38 mW	Intelligent agriculture
Dang et al.^306^	2022	Wind cup	EMG & TENG	∼0.2 m	13 m/s	29.59 mW	Intelligent agriculture
Wang et al.^307^	2022	Wind cup	EMG & TENG	∼0.14 m	7 m/s	8.29 mW	Intelligent agriculture
Sardini et al.^310^	2010	Horizontal	EMG	65 mm	45 m/s	9 mW	Wind sensing
Tan et al.^311^	2011	Horizontal	EMG	60 mm	3.62 m/s	13 mW	Wind sensing
Wang et al.^312^	2018	Wind cup	EMG & TENG	∼60 mm	1,000 rpm	438.9 mW/kg	Wind sensing
Fan et al.^313^	2020	Wind cup	EMG & TENG	170 mm	9 m/s	18.96 mW	Wind sensing
Zhao et al.^315^	2022	Savonius	TENG	–	15 m/s	11.7 mW/m^2^	Wind sensing
Zhu et al.^317^	2022	Wind mill	TENG	∼130 mm	1.65 m/s	0.753 μW	Wind sensing

Research on SWT design and optimization primarily aims to augment WER in complex wind environments and meet high power requirements in urban and transportation domains. To achieve superior system performance and stability, rotor structure and layout optimization are key aspects when catering to complex applications or varying wind conditions. SWT structures that utilize EMG and fit within limited size constraints have become a prominent medium for energy transfer, delivering stable and powerful output. On the other hand, in low-power applications such as agriculture or wind monitoring, researchers prefer designing SWTs that prioritize environmental adaptability and reliability over output performance, with simple rotors or wind cups becoming mainstream to provide WER. Researchers invest in optimizing PGMs, choosing TENGs in particular for their high output voltage and potential to act as self-powered wind sensors. PEGs, due to their complex conversion structures, seem to hold less favor in practical SWT applications, which prioritize feasibility and stability over scientific exploration.

In Table 3, the performances of SWTs reported in the literature are exhibited. A plethora of different performance metrics have been used by researchers, creating challenges while comparing the efficacy of the SWT designs. At the macroscopic level, in high wind environments, the classical rotor is widely preferred, allowing EMGs to achieve above 0.1 W output. At the microscopic level, TENGs are favored for their excellent output performance, while for practical applications, simple rotors or wind cups may be more relevant, due to limitations in aerodynamic performance from the ruler shrinkage effect. SWTs' energy conversion efficiency, weakly reported in applied studies and generally lower than the theoretical analysis, should be improved, indicating the potential for further research in this area. Furthermore, few practical applications of SWTs have been established to power distributed applications, with most of the current work limited to laboratory validation. This highlights the need for more research to bridge the gap between laboratory and commercial applications.

## Research trends, gaps, and future directions

### Research trends

A comprehensive analysis of the published literature on SWT was performed by searching for relevant topics in the Web of Science (WoS) international academic paper search engine database. To ensure the quality of the literature, the data range only covered the WoS core collection, and the time frame was restricted to 2012–2022.

**Figure 18:**
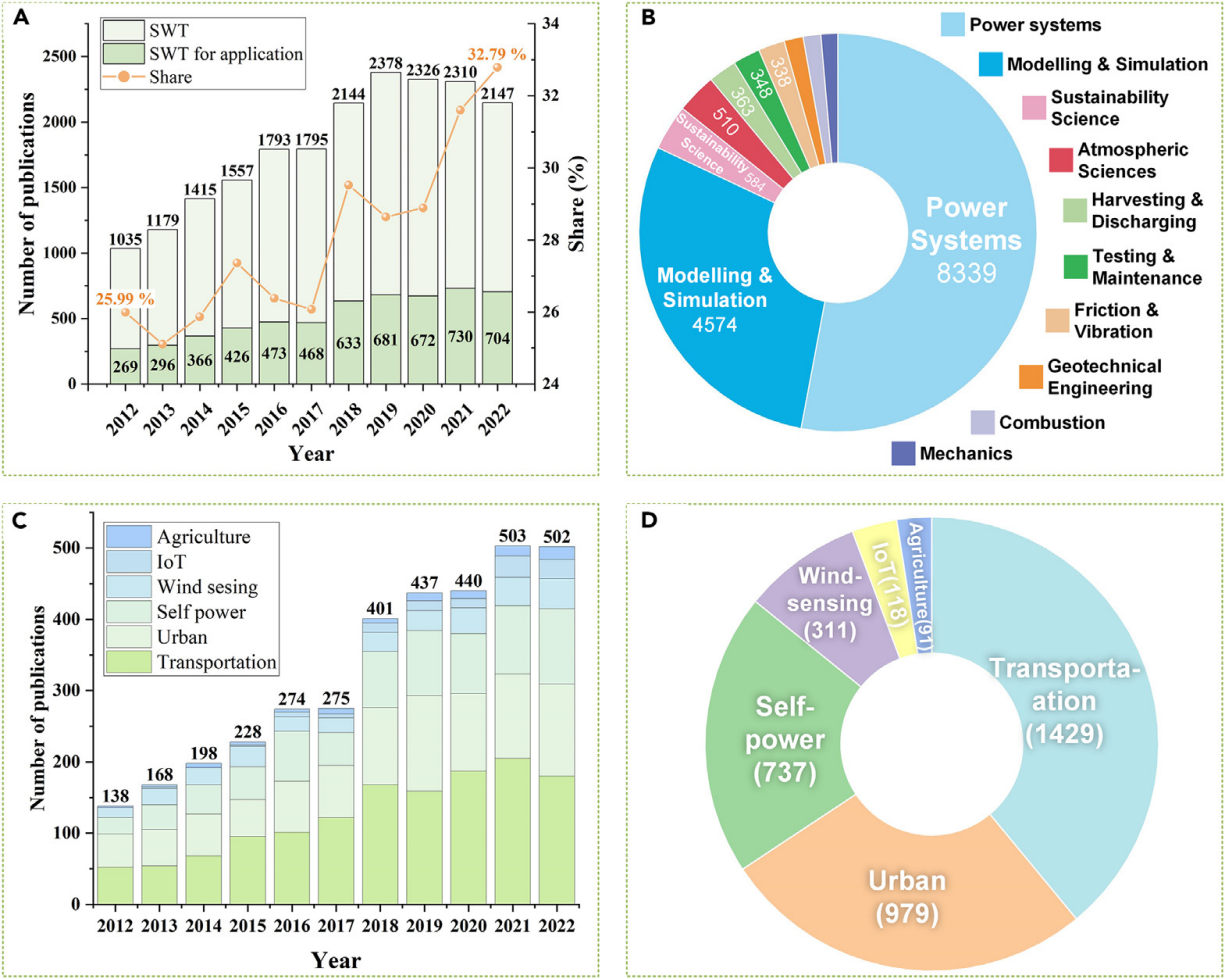
Analysis of research trends in the published literature (A) Trends in annual publication numbers. (B) The top ten topics covered by SWT. (C) Trends of SWT for IoT applications. (D) Share of each application field.

Figure 18A portrays the recent development trends in the field of SWT and its applications. It is noteworthy that there has been a surge in SWT research, with an increase in publications from 1,035 publications per year in 2012 to over 2,300 publications in recent years. In addition, research on SWT applications has seen significant development, with the number of published papers increasing by 2.7 times, from 269 per year in 2012 to 730 in 2021. As research advances, more researchers have shifted their focus to applying SWTs, resulting in an increase in the proportion of application research from 25.99% to 32.79%. In Figure 18B, the top ten themes in the field of SWT are presented, highlighting the diverse range of research conducted under the main themes of "Power Systems" and "Modeling & Simulation." The growth was seen in publications and the variety of macroscopic themes in this field indicate that SWTs have gained the attention of researchers across multiple scientific disciplines.

Development trends of IoT-based SWT sub-domains were analyzed using topic-based searches, illustrated in Figures 18C and 18D. SWTs play a critical role in addressing power-related challenges for IoT devices, particularly in the transportation (1,429 publications) and urban environment (979 publications) domains. Research on the IoT but not tied to specific applications was identified by searching topic words like "self-power" and "IoT." A considerable number of research articles attest to the significant potential of IoT-based SWTs for practical applications. Wind sensing (311 publications) is a crucial application that enables SWTs to be used not only as an energy supply for IoT but also in monitoring systems. Although smart agriculture is still emerging in the IoT era, with a comparatively lower number of publications, the trend is increasing, indicating its potential for substantial growth, as it proves to be a driving force for IoT technology in traditional fields.

### Research gaps and future directions

**Figure 19:**
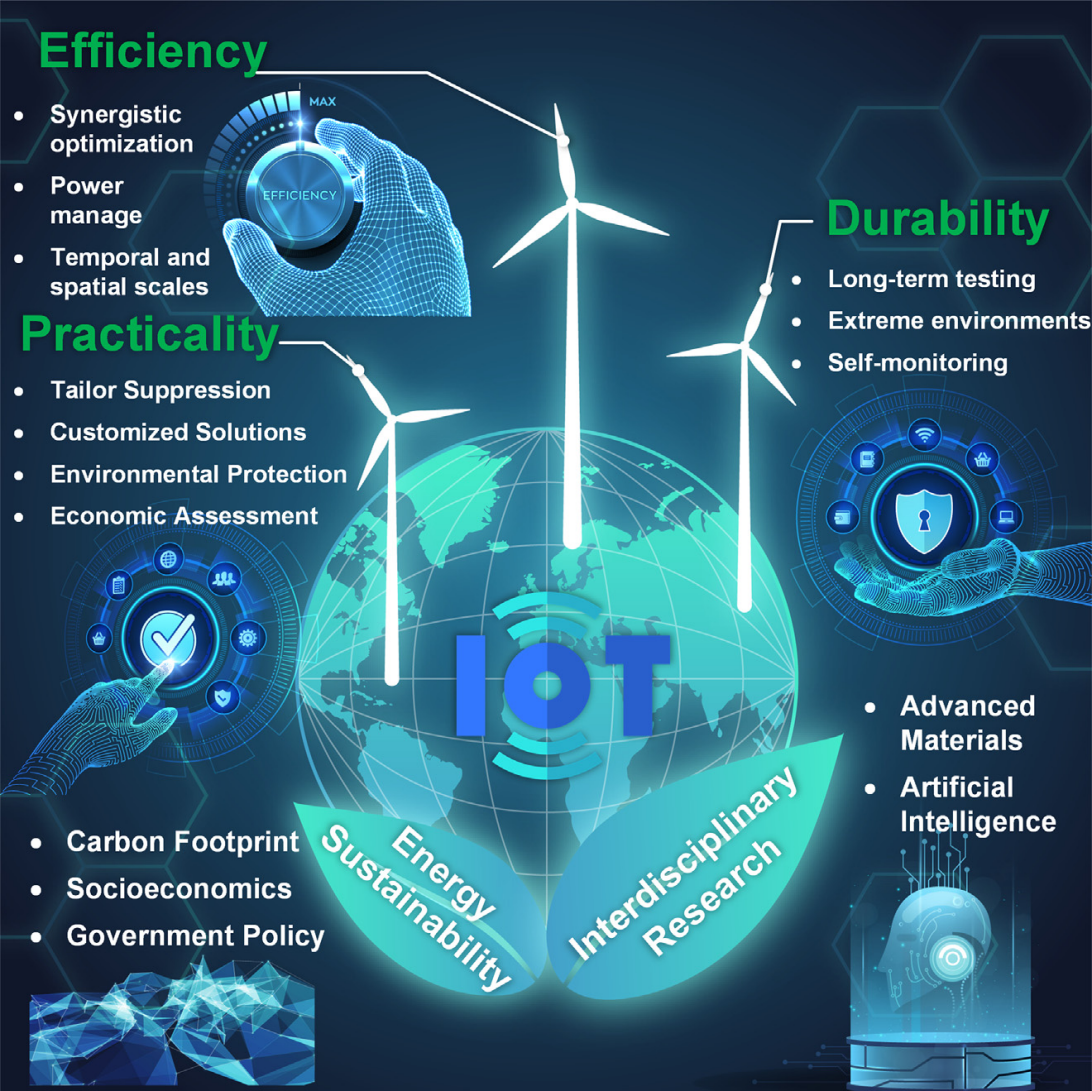
The future roadmap of SWT in IoT applications

This section presents an overview of the research gaps and future directions in SWT for IoT applications, including power generation performance, practicality, and durability, as shown in Figure 19 and Table 4. Despite the promising development of SWT for IoT applications, there are still challenges and difficulties to be overcome during commercialization.

**Table 4:** Research gaps and future directions of SWT for distributed applications

Keyword	Research Gap	Future Directions
Efficiency	The discrepancy between theoretical and actual power generation	• Synergistic system optimization study of WER-PGM-load • Energy storage system and power management strategy optimization research • Level of energy harvesting at temporal and spatial scales
Practicality	Existing small wind turbines have limitations in adaptability, feasibility, noise pollution, environmental damage, and economy	• Create practical and flexible solutions tailored to application-specific features • Investigate noise and vibration reduction techniques of SWT for IoT applications • Find ways to prevent harm or disruption to the environment and wildlife during product use • Perform a comprehensive evaluation of product functionality, dependability, and cost-effectiveness
Durability	Neglect of equipment reliability and maintenance in long-term and real wind conditions	• Further long-term performance tests under real wind conditions should be carried out • Consider the reliability of equipment in harsh and extreme environments • Research about self-monitoring and error diagnosis of equipment

The most critical factor for SWTs' widespread use in various IoT fields is practicality. Different application areas have different wind characteristics and load requirements, and the adaptability and feasibility of SWTs need to be adequately addressed to tailor solutions for the specific location. Factors to consider include wind speed range, equipment size, installation requirements, and portability. Furthermore, the noise annoyance caused by SWTs cannot be ignored, especially in urban environments or wildlife sanctuaries.^318^ Wind noise and the mechanical vibrations generated by SWTs inevitably waste energy that could be harnessed.^319^ Thus, there is a need to research noise reduction techniques and vibration suppression for SWTs and their clusters, enabling the smooth integration of SWTs for distributed applications into existing environments. In addition, the high-speed rotating blades of SWTs pose a danger to flying birds. Measures are necessary to make SWTs wildlife friendly, including color markings, acoustic warnings, and structural optimization of WER, such as bladeless rotors.^320^ On the other hand, economics is an important driver for SWT commercialization.^321^^,^^322^ Although SWTs and IoT applications promote sustainable energy development, their placement typically incurs additional costs. Current research has focused on the technical challenges of SWTs without much consideration of their techno-economic ratios. EMG technology is mature and commonly used. However, as SWTs become more compact, the need for processing precision and advanced materials may considerably increase production costs. For PEG and TENG, the development of high-performance and low-cost advanced materials is crucial for practical development. Besides performance studies, researchers need to consider the balance between performance and cost in an integrated manner to promote the SWT process.

SWTs are continuously exposed to harsh environments and frequent mechanical movements, making their durability of significant concern to researchers.^323^ At this stage of research, limited information is available on SWT testing in long-term and realistic wind conditions. Engineering validation is necessary to analyze the real-world performance, structural strength, and maintainability of SWTs in real-world applications. Some SWT applications are often located in harsh and extreme environments, and equipment stability of SWTs in extreme conditions should be considered fully. For instance, icing in severe cold environments can interfere with SWTs, particularly the blades.^324^ The formation of ice on the blades can reduce wind energy harvesting capabilities and shorten the system’s lifetime. Existing de-icing and anti-icing technologies are challenging to apply to SWTs in practice, and there is a need to develop specific anti-icing and de-icing technologies for SWTs in distributed applications. In addition, self-detection and diagnosis of equipment can aid in rapid fault location and maintenance management of SWT clusters, which can improve the stability of IoT applications and reduce maintenance costs.^325^

Interdisciplinary research plays an essential role in the future development of SWT. Advancements in materials technology,^326^ particularly metamaterials research,^327^ display tremendous potential in increasing the efficiency of SWT power generation, improving product stability, and reducing costs while increasing structural strength. With the development of micro and nanomanufacturing technology,^328^ EMG can perform micro-scale energy transfer while the performance of PEG and TENG will significantly increase with the development of advanced material technology, reducing their manufacturing cost and increasing practicality. Metamaterials research will also enhance the structural strength of micro and nano-scale SWTs, extend their service life, and broaden their working range and environmental conditions. The rise of AI technology has given a tremendous boost to SWT research.^163^^,^^329^ AI technology has great potential in the coupled optimization study of "Wind-SWT-Load-Monitoring," thereby allowing the SWT system to become miniaturized, multi-functional, and integrated. This development provides an exciting energy supply and self-sensing mechanisms for wind energy harvesting and IoT.

In addition to the technical challenges, the influence of carbon footprint, social economy, and policy on sustainable IoT networks based on SWTs cannot be overlooked. With significant advancements in computing, networking, and sensing technologies, sustainable IoT has emerged as a critical element in the functioning of modern societies. To evaluate the environmental impact of a sustainable IoT system, it is essential to adopt a life cycle assessment approach that considers the energy and material flow throughout its lifespan. Furthermore, the ecosystem’s impact should not be disregarded, necessitating the use of renewable resources during the production process to reduce carbon footprints and production costs. These factors must be thoroughly considered during the commercialization process to ensure global accessibility to these devices, enabling countries to achieve their climate and energy sustainability goals. It is crucial to conduct a comprehensive socio-economic assessment of sustainable IoT that accommodates the diverse geographic information, social conditions, and economic levels of various regions. The rapid expansion of sustainable IoT brings forth unexpected and significant environmental consequences, underscoring the importance of efficient management of equipment production, usage, and end-of-life processes. It is necessary to establish localized usage and management standards based on specific conditions and promote orderly commercialization and development of sustainable IoT through legislation, policies, guidelines, and similar measures. This approach strikes a balance between application value and environmental impact, fostering an eco-friendly development of sustainable IoT and ultimately achieving sustainable development goals.

## Conclusions

SWTs have gained significant attention in the past decade as achieving climate goals and energy sustainability remains a top priority, while the demand for IoT increases. This paper offers a comprehensive review of the status and recent advancements in research on SWTs for IoT applications, notably WERs, power generator systems, and IoT applications. It begins by describing and comparing the working principles and characteristics of various technical solutions, as well as reviewing their recent progress and representative works. Afterward, IoT application scenarios and their recent advancements are summarized. Furthermore, future research directions and the vast development potential offered by the development of interdisciplinary technologies are pointed out based on the analysis of existing research gaps.

WERs convert flowing wind energy into rotating mechanical energy. This process usually involves different types of rotors, including the horizontal rotor, Darrieus rotor, and Savonius rotor. While horizontal and Darrieus rotors tend to offer better harvesting potential due to their use of airflow lift, the simple structure and excellent self-starting performance of Savonius rotors are equally vital in some applications. Wind energy harvesting ability is currently a significant research objective in academia, and many innovative technologies have been developed to improve rotor starting and operational performance, notably blade design, rotor optimization, and additional configurations.

There are typically three generator mechanisms that are applicable to SWTs, namely, electromagnetic generators, piezoelectric generators, and TENGs. Each PGM exhibits unique characteristics and has been utilized in suitable WERs with varying structures and sizes. EMGs display high current and low voltage output, with performance dependent upon the rate of flux change. As a result, they are ideal for operation at high wind speeds or high rotational speeds. Currently, the limitation of EMGs' performance in SWT applications caused by the ruler scaling effect is a crucial challenge to be addressed. Meanwhile, PEGs demonstrate balanced performance but possess a low operating bandwidth. Piezoelectric topology, on the other hand, cannot directly couple the rotor, making the design of a suitable exciter a primary research effort. Finally, TENGs sustain significantly decreased lifetimes when operating at high frequency and are most suitable for low-wind-speed environments. In conclusion, the design of PGMs for SWTs is contingent on the adopted WER, input frequency, and application target.

Major and promising IoT application areas are summarized, including transportation, urban environment, intelligent agriculture, and self-powered wind sensing. As various IoT wind energy applications require different WERs, PGMs, architectures, and performance, it is necessary to design and optimize SWT systems to suit each specific application and its corresponding technical requirements. However, this process presents a challenge as environmental characteristics and corresponding technical solutions tend to vary significantly. Owing to differences in application areas and research methods, it is challenging to fully evaluate and compare the performance of SWTs objectively. The limited number of reported practical application examples of SWTs powering distributed applications indicates the need for more work to improve practical performance.

Despite the promising developments of SWTs for IoT applications, there remain challenges and difficulties to be overcome in their commercial implementation. These factors include efficiency, practicality, and durability. To address these challenges, researchers must expand their focus beyond SWT performance in the laboratory or on a computer and increase their efforts in collaborative optimization, power management, energy forecasting, feasibility studies, environmental protection, techno-economic analysis, reliability, and self-monitoring. Moreover, emerging interdisciplinary research, such as advanced materials technology and AI, will play a critical role in the future development of SWT. The impact of carbon footprint, socioeconomics, and policies on sustainable IoT networks based on SWTs is of paramount importance. A comprehensive approach encompassing integrated assessments and management of equipment production, usage, and end-of-life processes becomes imperative to ensure global accessibility to these devices and enable countries to achieve their climate and energy sustainability objectives. We anticipate that SWTs for IoT applications will remain a popular research topic, and the next decade will witness rapid growth in SWTs' various IoT applications.
